# Single-cell transcriptomic analysis of adult mouse pituitary reveals sexual dimorphism and physiologic demand-induced cellular plasticity

**DOI:** 10.1007/s13238-020-00705-x

**Published:** 2020-03-19

**Authors:** Yugong Ho, Peng Hu, Michael T. Peel, Sixing Chen, Pablo G. Camara, Douglas J. Epstein, Hao Wu, Stephen A. Liebhaber

**Affiliations:** 1 Departments of Genetics, Perelman School of Medicine, University of Pennsylvania, Philadelphia, PA 19104 USA; 2 Departments of Medicine, Perelman School of Medicine, University of Pennsylvania, Philadelphia, PA 19104 USA; 3 Penn Epigenetics Institute, Perelman School of Medicine, University of Pennsylvania, Philadelphia, PA 19104 USA; 4 Penn Institute for Biomedical Informatics, Perelman School of Medicine, University of Pennsylvania, Philadelphia, PA 19104 USA

**Keywords:** mouse pituitary, cellular plasticity, sexual dimorphism, single-cell RNA sequencing

## Abstract

The anterior pituitary gland drives highly conserved physiologic processes in mammalian species. These hormonally controlled processes are central to somatic growth, pubertal transformation, fertility, lactation, and metabolism. Current cellular models of mammalian anteiror pituitary, largely built on candidate gene based immuno-histochemical and mRNA analyses, suggest that each of the seven hormones synthesized by the pituitary is produced by a specific and exclusive cell lineage. However, emerging evidence suggests more complex relationship between hormone specificity and cell plasticity. Here we have applied massively parallel single-cell RNA sequencing (scRNA-seq), in conjunction with complementary imaging-based single-cell analyses of mRNAs and proteins, to systematically map both cell-type diversity and functional state heterogeneity in adult male and female mouse pituitaries at single-cell resolution and in the context of major physiologic demands. These quantitative single-cell analyses reveal sex-specific cell-type composition under normal pituitary homeostasis, identify an array of cells associated with complex complements of hormone-enrichment, and undercover non-hormone producing interstitial and supporting cell-types. Interestingly, we also identified a Pou1f1-expressing cell population that is characterized by a unique multi-hormone gene expression profile. In response to two well-defined physiologic stresses, dynamic shifts in cellular diversity and transcriptome profiles were observed for major hormone producing and the putative multi-hormone cells. These studies reveal unanticipated cellular complexity and plasticity in adult pituitary, and provide a rich resource for further validating and expanding our molecular understanding of pituitary gene expression programs and hormone production.

**Electronic supplementary material:**

The online version of this article (10.1007/s13238-020-00705-x) contains supplementary material, which is available to authorized users.

## Introduction

The pituitary is a key regulatory gland in mammals. Complex arrays of hormonal outputs from the pituitary play central roles in physiological pathways and a wide array of inherited and acquired pathological processes (Kelberman et al., 2009). These pathways impact post-natal growth, puberty, fertility, lactation, and metabolism. The pituitary contains a posterior lobe that comprises a direct extension of the central nervous system, and an anterior/median lobe (referred herein as anterior lobe) that is derived from oral ectoderm (Davis et al., 2013). The anterior pituitary contains cells that synthesize seven hormones: growth hormone (GH), prolactin (PRL), the β subunits of thyroid-stimulating hormone (TSHβ), luteinizing hormone β-subunit, (LHβ), and follicle-stimulating hormone β-subunit (FSHβ), as well as adrenocorticotrophic hormone (ACTH), and α melanocyte-stimulating hormone (α-MSH) (Fig. S1) (Zhu et al., 2007). Hormone synthesis and release from the anterior pituitary is coordinated by regulatory factors generated within signaling centers in the hypothalamus and transmitted to the pituitary via a dedicated hypothalamic-pituitary portal circulatory system (Vazquez-Borrego et al., 2018). A complete understanding of how these regulatory networks impact physiologic function requires unbiased and systematic analyses of cellular composition and relationships of cell lineages in the anterior pituitary as well as corresponding patterns of hormone gene expression.

The prevailing model of anterior pituitary structure and function is based predominantly on the assumption that each hormone is expressed from a discrete cell population (Zhu et al., 2007; Davis et al., 2013); i.e., seven hormones or hormone subunits are synthesized by a set of six distinct cell lineages (in this model the two gonadotrope hormone subunits, FSHβ and LHβ, are co-expressed from a single lineage) (Fig. S1). This discrete cellular model is primarily based on targeted immuno-histochemical and mRNA analyses of a handful marker genes. The varying sensitivities and specificities of these approaches (Nakane, 1970), their inductive nature, and their limited capacity to examine multiple hormonal expressions within single cells, suggest that the current model needs to be revisited by unbiased genome-wide approaches. In contrast, a more complex model of pituitary cell lineages and hormone expression has been suggested by previous reports of pituitary cells that express multiple hormones (Seuntjens et al., 2002a; Villalobos et al., 2004a, b). In addition, reports of pituitary cells with mitotic markers and factors linked to stem cell functions support complex models of pituitary dynamics linked to cellular expansion, *trans*-differentiation, and lineage plasticity (Andoniadou et al., 2013; Rizzoti et al., 2013; Cao et al., 2016). Thus, the current model of cell-type specific hormone expression in adult pituitary may not fully explain pituitary structure and functions in tissue homeostasis and the dynamic changes in hormone expression that occur in response to physiological stresses.

Recent advances in single cell technologies have enabled the comprehensive analysis of cell-type composition within a rapidly expanding array of tissues (Tanay and Regev, 2017). High-throughput single-cell RNA sequencing approaches, such as Drop-Seq and 10X Genomics platforms (Macosko et al., 2015; Zheng et al., 2017), can be used to effectively explore how defined cell lineages in various tissues respond to physiologic stresses and mediate specific functions. These comprehensive and unbiased approaches not only uncover cellular heterogeneity and novel cell types, but also reveal corresponding regulatory factors involved in lineage differentiation and function (Shekhar et al., 2016; Campbell et al., 2017; Chen et al., 2017; Hu et al., 2017).

In the current study, we have applied single cell RNA-seq methods (Macosko et al., 2015; Zheng et al., 2017), in conjunction with imaging-based *in situ* analysis of protein and RNA expression, to define the homeostatic as well as dynamic changes in cellular composition of the adult mouse anterior pituitary at single cell resolution. Our data are concordant with many aspects of the current model, most notably in the identification of individual cell clusters expressing specific pituitary hormones and the presence of sexual dimorphism in pituitary cell compositions. Interestingly, these data also reveal the presence of a putative population of multi-hormone expressing cells that can potentially contribute to the response of the pituitary to robust physiologic stresses linked to post-partum lactation and to stimulation by a hypothalamic regulatory factor. These analyses provide a comprehensive view of pituitary gene expression in adult pituitary and generate a rich resource for validating models of cell plasticity that underlie the capacity of the pituitary to respond to major physiologic demands.

## Results

### Comprehensive scRNA-seq analysis reveals both classical and less characterized hormone-producing cells

Studies of pituitary development and lineage differentiation have suggested a model in which each of six distinct hormone-producing cell-types expresses a corresponding polypeptide hormone (Fig. S1) (Zhu et al., 2007). The differentiation of these cells is controlled by transcription factors and signaling molecules (Kelberman et al., 2009). Multiple lines of genetic and biochemical evidence support that pituitary specific POU homeodomain transcription factor, POU1F1, serves a master regulator in driving terminal differentiation of cells expressing *Gh* (somatotropes), *Prl* (lactotropes), and *Tshb* (thyrotrope) (‘POU1F1-dependent lineages’; Fig. S1) (Camper et al., 1990; Li et al., 1990). The most compelling support for this function is the observation that loss of *Pou1f1* gene expression results in the combined loss of *Gh*, *Prl*, and *Tshb* gene expression in both mice (Camper et al., 1990; Li et al., 1990) and humans (Ohta et al., 1992; Radovick et al., 1992).

**Figure 1:**
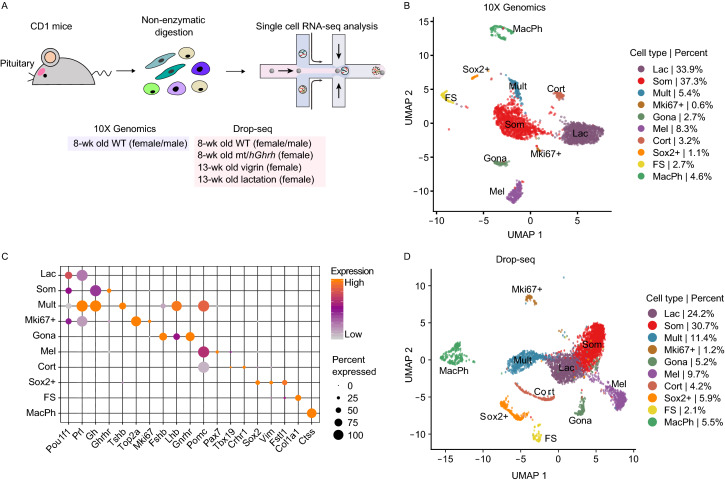
**Single cell transcriptome analysis of the adult mouse pituitary**. (A) Overview. The diagram summarizes the process of cell isolation and single cell RNA-seq analysis of the mouse pituitary using each of 2 platforms: 10X Genomics and Drop-seq. (B) Uniform manifold approximation and projection (UMAP) visualization. 2,780 cells were analyzed by 10X Genomics platform from 8-week-old CD1 male and female mice (*n* = 1). Cell type was color-coded. (C) Dot plot of normalized expression level of representative markers of each cluster. (D) Uniform manifold approximation and projection (UMAP) visualization. 4,663 cells were analyzed by Drop-seq platform from 8-week-old CD1 male and female mice (*n* = 2). Cell type was color-coded

To explore the full spectrum of pituitary cell composition in an unbiased manner, we employed single-cell RNA-seq technology to analyze pituitaries harvested from different genders, ages, and physiologic conditions (a total of 10 independent analyses) using both commercial 10X Genomics and in-house Drop-seq platforms (Fig. 1A). After excluding cells of low sequencing complexity (See METHODS and Table S1), the transcriptomes of 21,185 cells were retained for downstream analysis. We first analyzed the cellular clusters in the cells from 7 to 8-week old, sexually naïve female and male mice captured by the 10X Genomics platform (Fig. 1B). Visualization of the data by Uniform Manifold Approximation and Projection (UMAP) (Stuart and Satija, 2019), revealed ten distinct clusters (Fig. 1B**)**.

Among the three major *Pou1f1*-expressing cell clusters (Fig. 1B and 1C), the largest of them was assigned as somatotropes (Som) based on the high level enrichment for both *Gh* and the cell surface receptor for the hypothalamic growth hormone releasing hormone (*Ghrhr*) (Fig. 1C) (Lin et al., 1993). The second *Pou1f1*^+^ cluster was identified as lactotropes (Lac) based on the high level expression of *Prl* mRNA (Fig. 1C) in conjunction with markers previously linked to *Prl* expression and lactotrope functions; glutamine receptors (*Gria2*, *Grik2*, and *Grik5*) involved in PRL hormone release (Durand et al., 2008), two transcriptional co-activators of estrogen receptor function (*Ddx5* and *Ddx17*) (Janknecht, 2010), and two transcription factors recently identified as enriched in *Prl*^+^ cells and implicated in *Prl* gene activation (*Nr4a1* and *Nr4a2)* (Table S2) (Peel et al., 2018). The third Pou1f1-expressing cluster (‘Mult’, as defined below) encompassed 5.4% of anterior pituitary cells from 7 to 8-week old CD1 mice (Fig. 1B) and was associated with a unique gene expression profile. First, we detected in this Mult cluster the co-expression of both *Gh* and *Prl* mRNAs at levels that were comparable to those detected in the somatotrope (Som) and lactotrope (Lac) clusters (Fig. 1C). Secondly, cells in this cluster were also highly enriched for mRNAs transcribed from the *Pomc* gene at levels comparable to that detected in the ‘Pou1f1-independent’ melanotrope and corticotrope lineages (Fig. 1C) (Zhu et al., 2007). Thirdly, substantial number of cells in this cluster also contained mRNAs encoding the gonadotrope hormone, *Lhb* (Fig. 1C). Lastly, 58.3% of the cells in the anterior pituitary that contained *Tshb* mRNA mapped to this cluster (Table S2). On the basis of these observations, we provisionally assigned this cluster as a ‘multi-hormone’ producing cluster (Mult). Further analysis of the differentially expressed genes revealed that several genes involved in metabolism (*Gpx3*, *Ddah1*, and *Nme1*) were exclusively enriched in this cluster (Table S2).

The scRNA-seq analysis revealed a fourth *Pou1f1*^+^ cell cluster (“Mki67+” in Fig. 1B) that expresses proliferating cell markers such as *Top2a* and *Mki67* (Fig. 1C). This observation is consistent with prior reports that the majority of proliferating cells in the adult pituitary are positive for *Pou1f1* expression (Zhu et al., 2015; Cao et al., 2016). Notably, compared to “Som” and “Lac” clusters in Fig. 1B, both “Mult” and “Mki67+” clusters are associated with lower level of Pou1f1 mRNAs, indicating the potentially distinct regulatory functions of POU1F1 in these two populations of cells. Collectively, the single cell analysis of the adult pituitary not only identified expected *Pou1f1*^+^ populations of somatotropes and lactotropes, but also a novel *Pou1f1*^+^ cluster containing cells with a complex pattern of multi-hormone gene expression.

Three of remaining six cell clusters could be directly assigned to specific hormone-expressing cell-types based on patterns of marker gene expression (Fig. 1B and 1C). A cluster corresponding to melanotropes (‘Mel’) was assigned based on the co-expression of pro-opiomelanocortin (*Pomc)* prohormone mRNA, the transcription factor *Tbx19*, and the melanotrope-restricted paired homeodomain transcription factor *Pax7* (Fig. 1C) (Budry et al., 2012; Mayran et al., 2018). A second cluster was assigned as a corticotrope cell cluster (‘Cort’). These cells shared with the melanotrope cluster in enrichment for *Pomc* and *Tbx19* mRNAs but lacked substantial level of *Pax7* mRNA (Fig. 1C) (Philips et al., 1997; Lamolet et al., 2001; Liu et al., 2006). A third *Pou1f1*-independent cluster was annotated as gonadotropes (‘Gona’ cluster) based on the enrichment for mRNAs encoding the two gonadotrope-specific hormone subunits, *Fshb* and *Lhb*, in conjunction with the gonadotrope-restricted cell surface receptor for the hypothalamic regulatory factor, *Gnrhr* (Fig. 1C) (Ingraham et al., 1994; Zhu et al., 2007). In summary, our unbiased scRNA-seq analysis of the adult mouse pituitary identified a set of *Pou1f1*-enriched clusters and a set of clusters corresponding to three *Pou1f1*-independent lineages (melanotropes, corticotropes, and gonadotropes).

### Single cell RNA-seq identifies non-hormonal cell clusters in the adult pituitary

The scRNA-seq data sets identified three additional clusters that represented non-hormonal cell lineages. One of these clusters was identified as folliculostellate (‘FS’ cluster in Fig. 1B) cells based on known marker genes (Table S2). FS cells have been proposed to serve a variety of structural, paracrine, and/or support functions in the pituitary (Nakajima et al., 1980; Theogaraj et al., 2005; Devnath and Inoue, 2008).

The second cluster among the non-hormonal lineages, representing 1.1% of the cell in the analysis, was assigned as a putative stem cell cluster (‘Sox2+^’^ clusters; Fig. 1B). This assignment was based on the expression of the progenitor/stem marker *Sox2* (Fig. 1C) (Andoniadou et al., 2013; Rizzoti et al., 2013). A separate cluster was identified as macrophage (‘MacPh’ cluster) based on the expression of the known macrophage marker gene, *C1qa* (Zahuczky et al., 2011) (Fig. 1B). In summary, these data reveal three cell clusters in the pituitary that serve functions other than direct production of polypeptide hormones.

### Cross-platform scRNA-seq analysis

To assess platform-specific bias in our study, we compared the 10X Genomic platform analysis (above) with a transcriptome profile of 18,405 cells using the Drop-seq platform (cells isolated from four 7 to 8- week old, sexually naïve CD mice, three lactating female mice, one 13 week old, sexually naïve female control, and three *hGhrh* transgenic female mice) (Zheng et al., 2017; Cheung et al., 2018) (See Table S1 and Fig. S3). The analysis using the Drop-seq platform identified all of the cellular clusters identified using the 10X Genomics platform (Fig. 1D). The correlation analysis revealed that the clustering was highly conserved using these two different platforms (Fig. S2B). These results support the technical robustness and biological reproducibility of our scRNA-seq analysis.

We then compared our pituitary scRNA-seq analysis with a published pituitary scRNA-seq data set (Cheung et al., 2018) by computing the pairwise Pearson correlation coefficients between each pair of cell types for cluster marker genes. We compared the transcriptomes of the male mice in our 10X Genomics’ dataset with the published dataset which was limited to analysis of male mice using the 10X Genomics platform (Cheung et al., 2018). Our analysis of this published dataset identified two sub-clusters of lactotropes and three sub-clusters of somatotropes in (Fig. S2C). The comparison of the transcriptomic profiles of the two different datasets revealed that these two datasets are highly comparable in the cell clustering (Fig. S2D). Although the multi-hormone cluster was not specifically identified in the published report, we observed that the sub-cluster 1 of the somatotropes (Som1) was highly correlated with the multi-hormone cluster in our dataset (Fig. S2D). Taken together, these results show that our scRNA-seq analysis was largely consistent with the results from previous findings and that the putative multi-hormone cluster can be identified irrespective of scRNA-seq platforms.

### Sexual dimorphism in the organization and composition in the mouse pituitary

**Figure 2:**
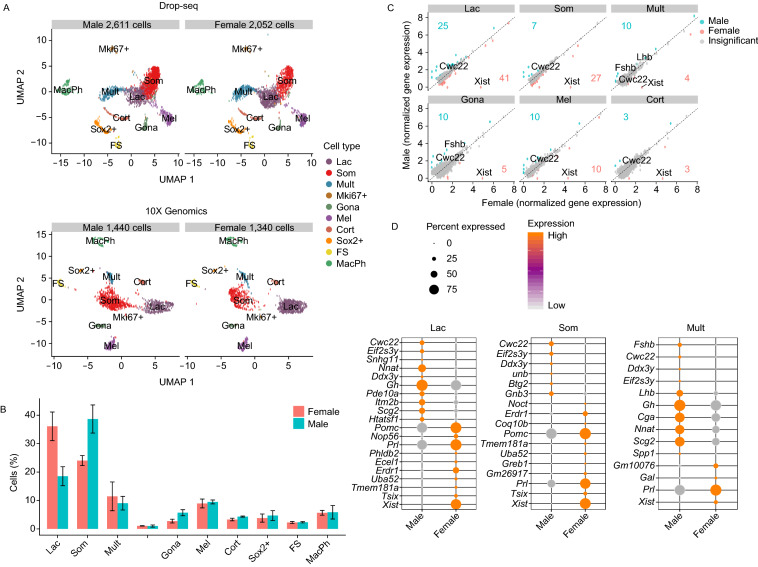
**Cell-type-specific sexual dimorphism of gene expression in pituitary cells**. (A) UMAP visualization of 8-week-old CD1 male and female pituitary cells. Cells were analyzed by two platforms: Drop-seq (top) and 10X Genomics (bottom). (B) Cell type composition between male and female mice (*n* = 3, 1 from 10X Genomics; 2 from Drop-seq). (C) Scatter plot comparing the gene expression levels of male and female pituitary cells across cell types. Differentially expressed genes (log FC > 0.1, adjust *P* value < 0.05) were color-coded; male (blue) and female (red). Sex specific genes (*Xist*, *Cwc22*), *Fshb* and *Lhb* were highlighted. The numbers in each frame represent the numbers of the differentially expressed genes between different genders. (D) Dot plot of normalized expression level of representative differentially expressed genes in Lac, Som and Mult clusters

Synthesis and secretion of the pituitary hormones are impacted by multiple gender-specific developmental and physiologic stimuli. These controls drive critical somatic alterations linked to puberty, reproductive cycling, pregnancy, and lactation. The degree to which these gender-specific functions are linked to differences in the cellular composition of the male and female pituitaries remains poorly defined (Lamberts and Macleod, 1990; Nishida et al., 2005). To address the issue of sexual dimorphism at the single-cell level, we directly compared the transcriptomes of cells isolated from 7 to 8- week old sexually naïve male and female pituitaries in our scRNA-seq datasets (Fig. 2A). Gender-specific analysis of major *Pou1f1*^+^ clusters revealed a relative predominance of somatotropes over lactotropes in males with a reciprocal enrichment of lactotropes over somatotropes in females (Fig. 2B).

Marked sexual dimorphism was also evident in the gonadotrope cluster. This cluster constituted a substantially larger fraction of the pituitary cell population in male compared with female mice (6.5% vs. 3.3% of total pituitary cells, respectively) (Fig. 2B). This dimorphism in gonadotrope cluster size is consistent with previously reported higher serum levels of FSHβ in male versus female mice (Michael et al., 1980). In contrast to the predominant expression of *Fshb* mRNA in males, the expression of *Lhb* was equivalent between males and females in gonadotropes (Fig. 2C). These data lead us to conclude that relative representations of somatotropes and lactotropes populations differ between the two genders and that the two gonadotrope hormones, *Lhb* and *Fshb*, are differentially expressed in the male and female pituitaries.

The analysis of the differential gene expression between males and females in Lac and Mult cluster revealed that *Secretogranin II* (*Scg2*), encoding a protein involved in regulating the biogenesis of secretory granules, is enriched in male and *Secreted Phosphoprotein 1* (*Spp1*), encoding a protein involved in the gonadotrope-releasing hormone (GnRH) signaling pathway was enriched in the Mult cluster in male (Fig. 2D). These data revealed the genes involved in secretory function were differentially expressed in a gender-specific manner.

### Single cell mRNA and protein analyses validate the findings in single cell transcriptomic analysis

**Figure 3:**
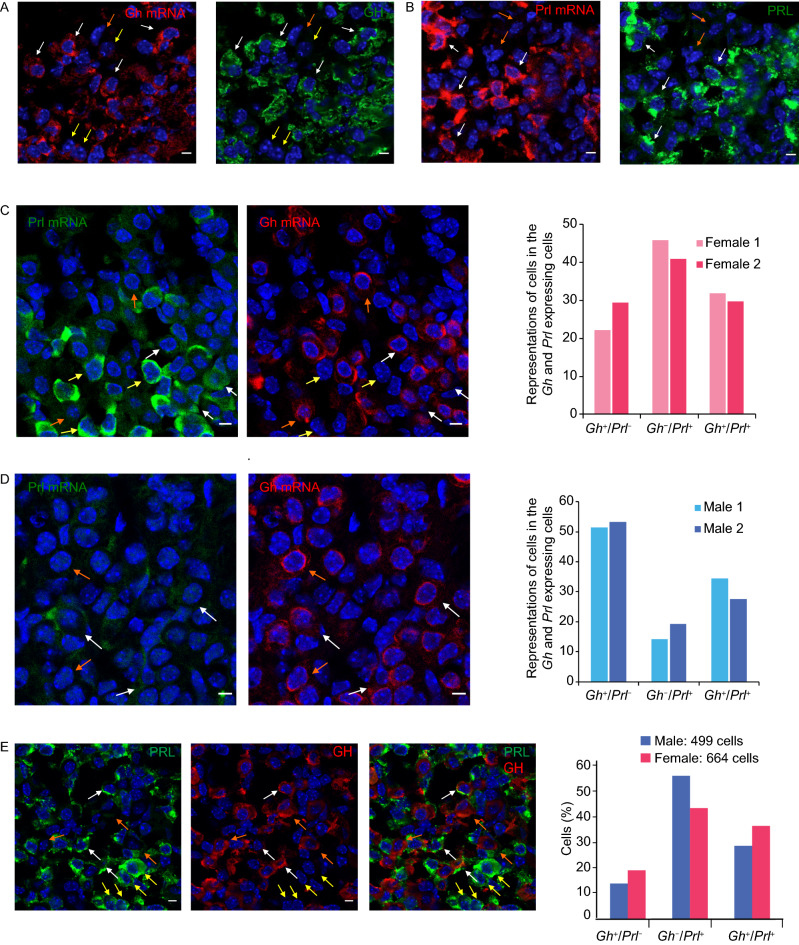
**Combined single cell RNA fluorescent*in situ*hybridization (scRNA FISH) and IF analyses in tissue sections detect frequent co-expression of*Gh*and*Prl*and sexually dimorphic expression of hormonal genes in the mouse pituitary**. (A) Correlation of *Gh* mRNA and GH protein detection. Combined single cell RNA FISH and IF analyses (scRNA FISH/IF) were performed in adult pituitaries (See METHODS). Left panel: detection of *Gh* mRNA by scRNA FISH. Scale bar = 5 μm. Right panel: detection of GH proteins by IF analysis in the same field. Scale bar: 5 μm. The white arrows indicate the cells with robust signals for both *Gh* mRNA and GH protein. The yellow arrows indicated cells with trace levels of *Gh* mRNA and lack of GH protein signal. The orange arrow indicates a cell lacking *Gh* mRNA and GH protein signals. (B) Correlation of *Prl* mRNA and PRL protein detection. Combined single cell RNA FISH and IF analyses (scRNA FISH/IF) were performed in female adult pituitaries (See METHODS). Left panel: detection of *Prl* mRNA by scRNA FISH analysis. Scale bar = 5 μm. Right panel: detection of PRL protein by IF analysis in the same field. Scale bar: 5 μm. The white arrows indicate four cells with robust signals for both *Prl* mRNA and PRL protein. The orange arrows indicate two cells lacking *Prl* mRNA and PRL protein signals. (C) ScRNA FISH analysis of female adult mouse pituitary tissue sections: concordance of *Gh* with *Prl* expression *in vivo*. Left panel: *Prl* mRNA detection. *Prl* mRNAs were detected by FISH using an array of anti-sense oligos (See METHODS). Bars = 5 μm. Right panel: *Gh* mRNA detection. *GH* mRNA was detected by FISH using an array of anti-sense oligos (See METHODS). Bars = 5 μm The white arrows in the two frames indicate three examples of *Gh* and *Prl* dual positive cells. The orange arrows indicate two cells with *Gh* mRNA expression but without *Prl* mRNA expression. The yellow arrows indicate three cells with *Prl* mRNA expression but without *Gh* mRNA expression. Histogram: analysis of female pituitary cells for *Gh* and/or *Prl* mRNA. Pituitary tissue sections from 8-week old, sexually naïve adult female mice (*n* = 2) were analyzed by RNA FISH. Cells from 5 randomly selected images from each pituitary were analyzed (>600 cells per pituitary). The histogram displays the representation of cells positive for *Gh* but not *Prl* mRNA (*Gh*^+^*Prl*^*−*^; 27%), cells positive for *Prl* but not *Gh* Mrna (*Gh*^*−*^*Prl*^+^; 45.2%), and those dual positive cells (*Gh*^+^*Prl*^*−*^; 27.8%) in *Gh* and/or *Prl* expressing cells. (D) scRNA FISH analysis of male adult mouse pituitary tissue sections reveals a concordance of *Gh* with *Prl* expression *in vivo*. Left panel: *Prl* mRNA detection. *Prl* mRNAs were detected by FISH using an array of anti-sense oligos as described in Methods. Bars = 5 μm. Right panel: *Gh* mRNA detection. *GH* mRNA was detected by FISH using an array of anti-sense oligos as described in Methods. Bars = 5 μm. The white arrows indicate three representatives of the *Gh* and *Prl* dual positive cells. The orange arrows indicate two cells with *Gh* mRNA expression but without *Prl* mRNA expression. Histogram: analysis of male pituitary cells. Pituitary tissue sections from 8-week old, sexually naïve male mice (*n* = 2) were analyzed by scRNA FISH for *Gh* and *Prl* mRNA expression as described in (A) The histogram represents the percent of the total cell population positive for *Gh* but not *Prl* mRNA (*Gh*^+^*Prl*^*−*^; 52.3%), positive for *Prl* but not *Gh* mRNA (*Gh*^*−*^*Prl*^+^; 16.7%), and positive for both *Gh* and *Prl* mRNA (*Gh*^+^*Prl*^+^ 31%) in *Gh* and/or *Prl* expressing cells. (E) IF analysis performed on 8-week old female mice confirms co-expression of GH and PRL proteins in pituitary cells. Left panel: IF detection of PRL protein (green). The IF was. Scale bar = 5 μm. Middle panel: IF detection of GH protein (red) in the same field as in left panel. Scale bar = 5 μm. Right panel: The merged images of the PRL IF and GH IF. The white arrows indicate the cells with robust levels of GH protein and PRL protein. The yellow arrows indicate cells with robust level of PRL but without GH protein signal. The orange arrows indicate cells with robust level of GH protein but without PRL protein signal. Histogram: co-expression of GH and PRL in the pituitaries of male or female mice. The IF analysis was performed in pituitary tissue sections of one male mouse (499 GH and/or PRL expressing cells were analyzed) and one female mouse (664 GH and/or PRL expressing cells were analyzed)

The scRNA-seq analysis revealed that a subset set of cells in *Pou1f1*^+^ clusters of the 7 to 8-week old mice were positive for both *Gh* and *Prl* mRNAs (*Gh*^+^/*Prl*^+^). As an initial step in validating this prevalent co-expression of these two hormones, we next probed tissue sections and dissociated cells from adult male and female pituitaries by single cell RNA fluorescent in situ hybridization (scRNA-FISH) and immuno-fluorescent staining (IF). Pituitary tissue sections of 8-week old mice were hybridized with arrays of fluorescent oligo probes antisense to the *Prl* and *Gh* mRNAs and the GH and PRL proteins were detected by the corresponding antibodies (Figs. 3A and S4A). As expected, *Prl* and *Gh* expression was observed to be restricted to the anterior lobe (AL) while *Pomc* expression was detected in both the intermediate lobe (IL) and AL (Fig. S4A). All cells with *Gh* or *Prl* mRNA as visualized by scRNA FISH had a comparable IF signal for the corresponding proteins (Fig. 3A and 3B); when *Gh* or *Prl* mRNA was abundant the corresponding proteins were also abundant and when either mRNA was at trace levels or undetectable the corresponding protein was comparably trace or negative by IF (examples in Fig. 3A and 3B). In no case did we observe a substantial discordance between corresponding IF and scRNA-FISH signals.

The RNA-FISH analysis further confirmed that a fraction of the *Gh* and *Prl* expressing cells were dual positive for these two mRNAs in both female and male mice (Fig. 3C and 3D). The level of co-expression of *Gh* and *Prl* mRNAs was determined by analysis of 5 or more randomly selected fields (>600 cells per pituitary) of pituitary tissue sections. This analysis revealed that 30.8% of all the *Gh* and/or *Prl* expressing cells in the female pituitary co-expressed *Prl* and *Gh* mRNAs (*Gh*^+^/*Prl*^+^) while 25.8% were uniquely positive for *Gh* mRNA (*Gh*^+^/*Prl*^*−*^) and 43.4% cells were uniquely positive for *Prl* mRNA (Fig. 3C). A parallel study of pituitary cells from male mice revealed 31% of the cells as dual positive (*Gh*^+^/*Prl*^+^), 52.3% as uniquely *Gh* positive (*Gh*^+^/*Prl*^*−*^), and 16.7% as uniquely *Prl* mRNA positive (*Prl*^+^/*Gh*^*−*^) (Fig. 3D). We conclude from these studies that co-expression *Gh* and *Prl* mRNAs is prevalent in the *Pou1f1* cell population in the adult pituitary.

We next assessed the prevalence of GH and PRL co-expression at the protein level (Fig. 3E). A double IF for GH and PRL analysis was performed in the tissue sections of an 8-week old male mouse and an 8-week old female mouse. We observed that a subset of cells expressing either GH or PRL co-expressed both of the hormones (14% in the males and 19% in the females) (Fig. 3E). The difference in the percentages of dual positive cells detected by RNA-FISH and by IF most likely reflects the different sensitivities of these two approaches as indicated in our combined RNA-FISH and IF studies (Fig. 3).

We also investigated the gender-specific expression profiles of the *Fshb*. IF studies were used to define the composition of the FSHβ expression cells in male and female mice. Pituitary tissue sections of 8-week old sexually naïve mice were stained with antibody specific for FSHβ (Fig. S4B). The composition of the FSHβ expressing cells was calculated by analysis of >5 selected fields (>800 cells per pituitary) of pituitary tissue sections. The IF results revealed that the FSHβ expressing cells constituted 18.6% of cells in male pituitary and 8.8% in female pituitary (Fig. S4B). These results were consistent with the higher percentage of FSHβ expressing cells in males revealed in our single cell transcriptomic analysis (Fig. 2B and 2C).

**Figure 4:**
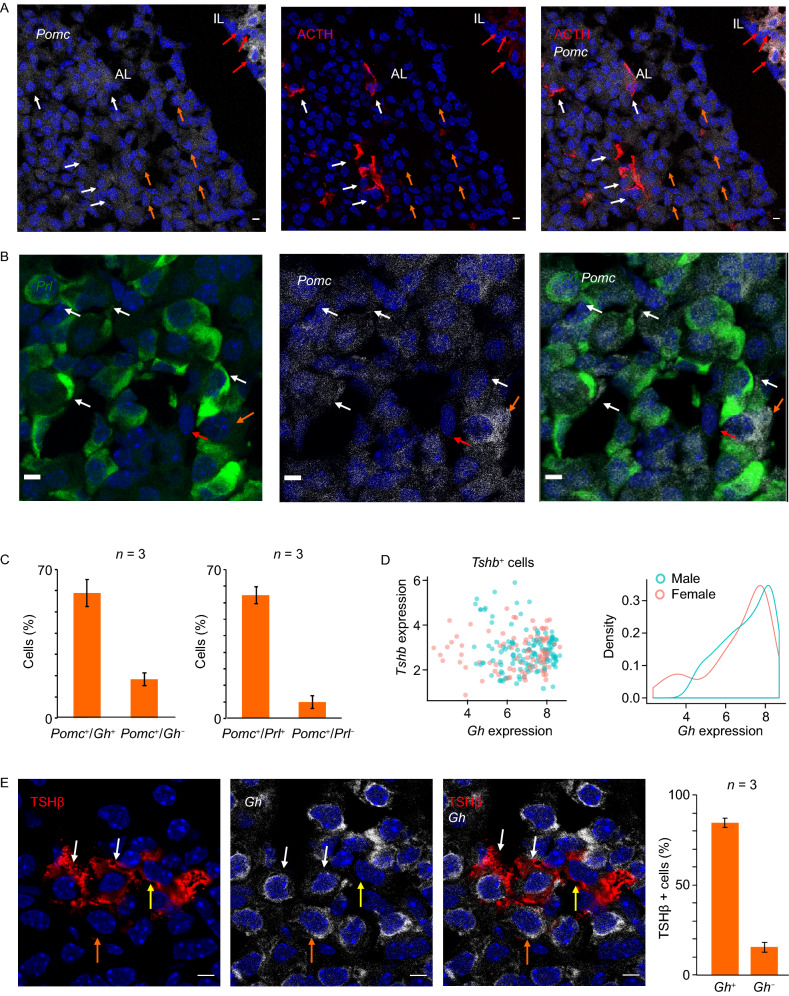
***Pomc*and*Tshb*mRNA is co-expressed with the*Gh*mRNA and*Prl*mRNA in the anterior lobe of the adult pituitary**. (A) *Pomc* mRNA is broadly expressed in the anterior lobe (AL) of the adult pituitary. Left panel: *Pomc* mRNA (gray) was detected by RNA FISH analysis using anti-sense oligo probes (See METHODS). Scale bar = 5 μm. Middle panel: ACTH protein (red) was detected by IF analysis using antibody against ACTH. The ACTH antibody also recognizes α-MSH, the peptide hormone generated by the cleavage of ACTH in the IL. Scale bar = 5 μm. Right panel: Merged images of the *Pomc* RNA FISH and ACTH IF in the same field. Scale bar = 5 μm. The white arrows indicate cells with *Pomc* mRNA and robust levels of ACTH protein in the AL. The orange arrows indicate cells with *Pomc* mRNA in non-corticotropes (ACTH negative) in AL. The merged images further confirm the robust expression of *Pomc* mRNA in the melanotropes (red arrows in the IL). (B) RNA FISH analysis detects co-expression of *Pomc* mRNA and *Prl* mRNA in cells of the anterior lobe of adult pituitary. *Prl* mRNA was detected by FISH using an array of 488-conjugated anti-sense oligonucleotide probes (green in left panel). *Pomc* mRNA was detected by FISH using an array of Cy5-conjugated anti-sense oligonucleotide probes (gray in middle panel). The right panel is the merged images of the *Prl* mRNA FISH and *Pomc* RNA FISH in the same field. The white arrows indicate cells with co-expression of *Prl* and *Pomc* mRNAs, the orange arrow indicates a cell with robust signal for *Pomc* mRNA and without *Prl* mRNA, and the red arrow indicates a cell that is negative for both *Prl* and *Pomc* mRNAs. Scale bar = 5 μm. (C) Histogram summarizes the number of *Pomc*^+^ cells that co-express *Prl* or *Gh* mRNAs in adult pituitary. Left graph: histogram summarizes the number of dual positive cells (*POMC*^+^*Prl*^+^; 74%) and cells positive for *Pomc* but not *Prl* mRNA (*POMC*^+^/*Prl*^*−*^; 9.8%). Right graph: histogram summarizes the number of dual positive cells (*POMC*^+^*Gh*^+^; 59%) and cells positive for *Pomc* but not *Gh* mRNA (*POMC*^+^/*Prl*^*−*^; 18.3%). (D) *Tshb* mRNA is predominantly co-expressed along with *Gh*. Left panel: 2D scatter plot of *Gh* in the *Tshb* mRNA expressing cells. Scatter plot showing the expression level of Tshb and Gh in Tshb+ cells of 8-week-old CD1 WT mice from Drop-seq data set. Sex was color-coded. Right panel: density plot of Gh expression level in Tshb+ cells. (E) Prevalence of *TSHβ* and *Gh* co-expression as revealed by combined RNA-FISH/IF. Detection of TSHβ protein (red in the left panel) was performed by IF and *Gh* RNA detection was established by scFISH (gray in the middle panel). The right panel is a merge of the RNAFISH and IF images. This single field analysis is representative of pituitary cells from tissue sections of three mice (two male mice and one female mouse). The white arrows indicate cells with robust expression of TSHβ protein and *Gh* mRNA. The orange arrow indicates a cell with robust expression of *Gh* mRNA but without TSHβ protein signal. The yellow arrow indicates a cell with TSHβ protein but without *Gh* mRNA signal. Nuclei were stained with DAPI (blue). Scale bar = 5 μm. Histogram: analysis of cells expressing TSHβ and *Gh* mRNAs. The RNA FISH/IF analysis was performed in three pituitaries (*n* = 3). The data reveals that 84.6% of the TSHβ protein positive cells co-expressed *Gh* mRNA

*Pomc* mRNA encodes a prohormone that generates multiple smaller peptide hormones by site-specific cleavages specific to particular cells in the pituitary (Cawley et al., 2016). Two of these peptides, ACTH and α-MSH, are lineage specific markers for anterior lobe (AL) corticotropes and intermediate lobe (IL) melanotropes, respectively (Zhu et al., 2007). The mapping of *Pomc* expression by scRNA-seq revealed that *Pomc* mRNA was present at high levels (equivalent to that detected in the corticotrope lineage) in the cells that constituted the multi-hormone cluster (Fig. 1C) and was co-expressed with *Gh* and *Prl* mRNAs. This observation was validated by scRNA FISH. Combined *Pomc* RNA-FISH and ACTH IF studies revealed that *Pomc* was also expressed in ACTH negative cells in the anterior pituitary (Fig. 4A). Combined RNA-FISH analyses of *Pomc* and *Prl* mRNAs revealed that majority of the *Prl* mRNA positive cells were also positive for *Pomc* mRNA (Fig. 4B). Remarkably, scRNA FISH analysis in female pituitary cells (*n* = 3 pituitaries) revealed that 74% of the cells co-expressed *Pomc* and *Prl* and only 9.8% of the cells were *Pomc*^+^ but *Prl*^*−*^ (Fig. 4C, left graph). A second scRNA FISH analysis in male pituitary cells (*n* = 3 pituitaries) revealed that 59% of all analyzed cells from male pituitary (*n* = 3 pituitaries) co-expressed both *Pomc* and *Gh* while only 18.3% of the cells were *Pomc*^*+*^ but *Gh*^*−*^ (Fig. 4B, right graph). These scRNA FISH and IF studies are concordant with the our scRNA-seq data and suggest that the *Pomc* gene is co-expressed with the *Gh* and/or *Prl* gene(s) in majority of cells in the AL of the adult mouse pituitary.

TSHβ is generally considered to constitute one of the three Pou1f1-dependent pituitary hormone genes (Fig. S1) and the expression of TSHβ is thought to define a distinct POU1F1-dependent ‘thyrotrope’ lineage (Zhu et al., 2007). While the scRNA-seq analysis defined clusters corresponding to the two major POU1F1-dependent lineages, somatotrope and lactotrope, as well clusters corresponding to all three of the POU1F1-independent lineages (corticotrope, melanotrope, and gonadotrope), the analysis failed to identify a discrete cell cluster that specifically corresponded to thyrotropes. Instead, *Tshβ*^+^ cells mapped to all three of the major ‘*Pou1f1* lineage’ clusters with a substantial representation in the multi-hormone cluster (Fig. 1C). A two-dimensional scatter plot of *Tshβ* and *Gh* mRNA distributions revealed that almost all of *Tshβ*^+^ cells co-expressed both *Gh* and/or *Prl* mRNAs (Fig. 4D). Further analysis revealed the concordance of *Tshβ* and *Gh* expression in the *Tshβ* expressing cells (Fig. 4D). This observation was validated by combining IF detection of TSHβ protein with scRNA FISH detection of *Gh* mRNA in male pituitaries; 84.6% of the TSHβ positive cells by IF were also positive for *Gh* mRNA by scRNA FISH (Fig. 4E). The co-expression of TSHβ and GH proteins was further confirmed by IF in cells from an 8-week WT CD1 male mouse; 61% % of TSHβ cells were also positive for GH protein (Fig. S4C). These orthogonal studies lead us conclude that *Tshb* expression, while demonstrating the expected localization to *Pou1f1*^+^ cells, does not define a distinct ‘thyrotrope’ cell cluster. Instead, we find that *Tshb* is expressed in conjunction with *Gh* and/or *Prl*, and maps predominantly to the multi-hormone cluster.

### The response of hormone-expressing clusters to major physiologic demands

**Figure 5:**
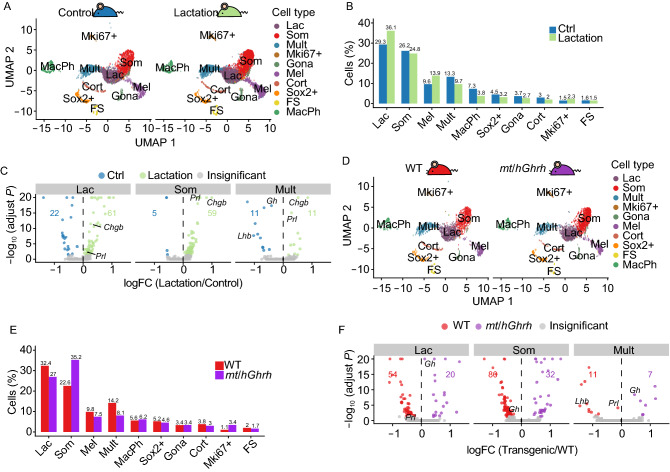
**Single cell analysis of hormone expressing clusters to physiologic demands**. (A) UMAP visualization of pituitary cells from 13-week-old virgin females and lactating females (Control mouse: *n* = 1 (3,532 cells); Lactating mouse: *n* = 2 (7,614 cells)). (B) Cell type composition of control and lactating females. (C) Volcano plot comparing the gene expression levels of control and lactating pituitary cells in Lac, Som and Mult clusters. (D) UMAP visualization of 8-week-old female pituitary cells in WT (*n* = 2 (2,052 cells) and *mt*/*Ghrh* mice (*n* = 1 (2,596 cells). (E) Cell type composition of the age and gender matched WT and *mt*/*hGhrh* mice. (F) Volcano plot comparing the gene expression levels of WT and *mt*/*hGhrh* pituitary cells across Lac, Som and Mult clusters

We next sought extend our analyses of pituitary cell populations to settings of defined physiologic stress. One of the most dramatic alterations in pituitary hormone production is the 10–15 fold increase in serum PRL levels during lactation in both mice and humans (Le Tissier et al., 2015). The molecular mechanisms underlying this increase are unclear (Castrique et al., 2010). In particular, it remains unclear whether this increase reflects an expansion of the lactotrope lineage and/or to alterations in hormone gene expression per lactotrope. To address this issue, we compared in two independent experiments the pituitary cell-type composition in three 13-week lactating female mice with an age-matched virgin female control (Rep 1 (one mouse) and Rep 2 (two mice)). This analysis revealed several noteworthy observations. First, the overall pattern of cell clustering in each of these 13-week females was similar to that of 7–8 weeks old mice (male and female) with the formation of a multi-hormone cluster in both studies (Fig. 5A). Second, the comparison between the lactating and the virgin 13-week females revealed an expansion of the lactotrope cluster in the lactating state (from 29.3% in control to 36.1% in lactating mice) with reciprocal decreases in representation of clusters representing multi-hormone, gonadotropes, corticotropes, and Sox2+ cells. In contrast to these shifts, the fraction of somatotropes remained largely unchanged (Fig. 5B). These data demonstrate substantial shifts in specific pituitary cell populations in the transition to the lactating state with a net increase in the representation of lactotropes.

We further identified the differentially expressed non-hormonal genes in lactating mice compared to control group. We noted a substantial increase in expression of the neuroendocrine vesicle secretory protein *Chgb* (Barbosa et al., 1991; Gill et al., 1991) (adjusted *P*-value = 3.36 × 10^−37^) (Fig. 5C). The increase in *Chgb* is consistent with the high demand for PRL secretion during lactation. These data lead us to conclude that the enhancement of PRL production during lactation in the mouse reflects the combined effects of an increase in the lactotrope population as well as an elevation in PRL secretion within the lactotrope cells.

In addition to its expression in lactotropes, *Prl* expression is also a prominent attribute of the multi-hormone cell cluster. We next sought to determine whether the cells in the multi-hormone cluster contributed to the increased pituitary production of PRL in support of lactation. We noted that the representation of the multi-hormone cluster in the lactating mice decreased from that in the control (13.3% vs. 9.7%, respectively) (Fig. 5B) while the expression of *Prl* in the cells within this cluster increased substantially (adjusted *P*-value = 8.42 × 10^−16^) (Fig. 5C). In contrast, the expression of *Lhb* in the cells within this cluster significantly decreased (adjusted *P*-value = 2.34 × 10^−8^). These shifts are consistent with prior reports that the LHβ secretion is decreased during pregnancy in parallel with the inhibition of estrous cycle (Smith and Fox, 1984). We also observed a highly significant increase of *Prl* expression (adjusted *P*-value = 9.11 × 10^−126^) in the somatotropes of lactating mice compared with the control (Fig. 5C). In summary, these results demonstrated that the cells in the multi-hormone and somatotrope clusters undergo transcriptomic shifts that support increased production of PRL in the lactating female. These findings lead us to conclude that the induction of *Prl* expression in lactating females is based on a complex mix of shifts in cell composition along with changes in cluster transcriptome profiles.

We next assessed the impact of a second major stress on the pituitary; the overexpression of *Ghrh*. GHRH is the major hypothalamic regulator of somatotrope function (Mayo et al., 1988); it is delivered to the anterior pituitary via the hypothalamic/pituitary venous portal circuit and binds to somatotrope cell surface receptors (GHRHR) to stimulate somatotropes proliferation of as well as to stimulate expression and secretion of GH (Mayo et al., 2000; Gaylinn, 2002). A well-described *mt*/*hGhrh* transgenic model (Mayo et al., 1988) has been previously reported and shown to drive high levels of GH expression in the mouse with resultant gigantism (Mayo et al., 1988; Ho et al., 2002). We performed Drop-Seq analysis of 2,596 pituitary cells isolated from two 8-week old virgin female mice carrying the *mt*/*hGhrh* transgene (Mayo et al., 1988) and age-matched virgin non-transgenic females. This analysis revealed the assembly of an array of clusters that paralleled those detected in 8-week old non-transgenic mice as well as the 13-week old virgin and lactating females (Fig. 5D). A multi-hormonal cluster was again observed in this analysis as well as the full array of hormone-expressing and non-hormonal cell clusters that were seen in the preceding analyses. When compared with the WT control, the analysis of the *hGhrh* transgenic pituitaries revealed a marked expansion in the somatotrope cluster; from 22.6% of total pituitary cells in WT to 35.2% in the *hGhrh* transgenic (Fig. 5E). This increase in the representation of the somatotrope cluster was paralleled by a reciprocal decrease in the representations of the multi-hormone (14.2% to 8.1%) and lactotrope (32.4% to 27%) clusters (Fig. 5E). Differential gene expression analysis in the *hGhrh*-transgenic pituitaries revealed significant increase in the level of *Gh* mRNA expression in cells within the lactotrope cluster (Fig. 5F). Importantly, *Gh* expression was also significantly increased in the multi-hormone cells with reciprocal decreases in *Prl*, *Lhb*, and *Pomc* mRNAs were (Fig. 5F). These shifts in cell cluster representation and gene expression in response to *Ghrh* overexpression highlight the contributions and coordination of the three major *Pou1f1*-expressing cell clusters in both their relative cell representations and in their gene expression profiles. Furthermore, GO enrichment analysis showed that the top 100 differentially expressed genes of the multi-hormone cells were enriched in hormone activity and secretory granule (Table S3). These studies further validated the utility of scRNA-seq analysis in identifying shifts in cell representation and transcriptome expression profiles in response to physiologic demands.

## Discussion

Current understanding of pituitary function is based upon a binary model in which each of the six distinct endocrine cell types is dedicated to the synthesis and secretion of its corresponding polypeptide hormone (Fig. S1). The function of each lineage is considered as mutually exclusive and under the control of a corresponding set of discrete hypothalamic/pituitary regulatory circuits (Zhu et al., 2007; Kelberman et al., 2009; Davis et al., 2013). However, scattered reports of pituitary cells co-expressing multiple hormones have suggested that the organization and function of the pituitary may be more complex than commonly appreciated (Frawley and Boockfor, 1991; Childs, 2000; Seuntjens et al., 2002a; Villalobos et al., 2004b) and the prevalence and complexity of ‘multi-hormone’ cells in the adult anterior pituitary has remained undefined on a systematic and global level. Thus, understanding the compositions and functions of pituitary cell lineages and their relationships to hormone expression warrants further study.

Here we report a series of orthogonal single-cell analyses that address fundamental aspects of pituitary cell organization, lineage specification, and functional plasticity. Our single cell transcriptome analyses yielded findings that were consistent with certain aspects of existing models while substantially extending and modifying others. While the analyses confirmed the presence of three discretely clustering *Pou1f1*-independent cell lineages (corticotropes, melanotropes, and gonadotropes (Fig. 1B)), they also revealed that the organization and functions of the Pou1f1-dependent lineages are substantially complex (Fig. 1). The initial presumption going into this study was that the clustering analysis would generate three discrete *Pou1f1*^+^ clusters corresponding to somatotropes, lactotropes, and thyrotropes, each dedicated to the exclusive expression of its corresponding hormone; *Gh*, *Prl*, and *Tshb*, respectively (Fig. S1) (Zhu et al., 2007). However, our unbiased scRNA-seq failed to confirm the existence of a unique ‘thyrotrope’ lineage and instead revealed that the majority of *Tshb* mRNA expression, along with a significant fraction of *Gh* and *Prl* mRNAs, were derived from a novel *Pou1f1*-expressing ‘multi-hormonal’ cell cluster. The discovery of this multi-hormone cell cluster challenges current models of pituitary lineage distinctions and the segregation of hormone expression.

Our multiple independent scRNA-seq analyses on pituitaries isolated from mice of varying ages and genders as well as under two well defined setting of physiologic stress reproducibly identified the unique multi-hormone cluster. Most intriguing was the observation that the cells in this cluster not only expressed well defined POU1F1-dependent genes such as *Gh*, *Prl*, and *Tshb*, but also expressed robust levels of mRNAs encoded by two genes, *Pomc* and *Lhb*, that are traditionally categorized as POU1F1-independent. A series of single cell RNA and protein imaging studies documented co-expression of the ‘POU1F1-dependent’ and the ‘POU1F1-independent’ hormone genes within individual cells (Figs. 2, 3 and S4).

While multi-hormone producing cells have been previously identified by others using immuno-fluorescent and targeted RT-PCR analyses (Seuntjens et al., 2002a, b; Villalobos et al., 2004a), their abundance, full array of hormone gene expression, and physiological function(s) have remained unclear. Our analyses reveal that the cells in this multi-hormone cluster share a defined transcription program and respond to physiological demands *in vivo*. For example, in lactating females, the level of *Prl* expression increases in the cells in this cluster with reciprocal decreases in *Lhb* and *Gh* mRNAs. Likewise, in transgenic mice overexpressing the *hGhrh* gene, *Gh* expression increases in the multi-hormone cells while the expression of other pituitary hormones significantly decreases. These observations suggest that cells in the multi-hormone cluster are able to respond to substantial physiological demands and exhibit plasticity of pituitary function and hormone production.

The multi-hormone cluster constitutes a substantial fraction (5.4%–11.4%) of total pituitary cell population. The cells within this cluster appear to serve as a transitional cell pool that can respond to shifts in hormone production. Such shifts are likely essential to support physiological demands imposed by pregnancy, lactation, and pubertal growth. Changes in hormone gene expression patterns in such a large number of cells can significantly facilitate the response of the hypothalamic/pituitary axis to acute physiologic demands. It will be of interest to further explore the molecular/signaling pathways that control the cellular plasticity of the cells within the multi-hormone cluster.

The developmental origin(s) of the *Pou1f1*^*+*^ multi-hormonal cells in adult mouse pituitary is presently unclear. The broad functional capacity of these cells cannot be accounted for in current models of anterior pituitary development and cellular differentiation (Fig. S1). However, recent studies suggest that these cells may be induced by the actions of PROP1. PROP1, a paired-like homeodomain transcription factor, was initially described as a factor that triggers the activation of *Pou1f1* and the differentiation of the POU1F1-dependent lineages during pituitary development (Andersen et al., 1995; Gage et al., 1996). However, recent lineage tracing studies in the mouse reveal that PROP1 is in fact expressed in progenitor cells that generate the full spectrum of endocrine cell types in the pituitary, those considered to be ‘POU1F1-dependent’ as well as those considered to be ‘POU1F1-independent’ (Davis et al., 2016; Perez Millan et al., 2016). Multi-hormone expressing cells may therefore be generated through the activity of PROP1 during pituitary development.

In addition to revealing the presence of the multi-hormone cell cluster, the Drop-seq analysis highlighted complex relationships between the functions of the other two major POU1F1 clusters, the somatotropes and lactotropes. While the cells in these two clusters could be readily identified by a preponderant expression of either *Gh* or *Prl* as well as by a number of corresponding signatory mRNAs (See Fig. 1 and accompanying text), the majority of these cells co-expressed *Gh* and *Prl* to varying degrees (Figs. 1 and 2). Furthermore, the levels of *Prl* expression in somatotropes increased substantially in lactating mice while *Gh* expression increased significantly in the lactotropes of the *hGhrh* transgenic mice (Fig. 5). These data suggest that the capacity of the somatotrope and lactotrope lineages to express both *Prl* and *Gh* may be critical to a robust response to major physiological demands.

The synthesis and secretion of the pituitary hormones are impacted by multiple physiologic variables that directly relate to sexual maturation, reproduction, and somatic development. As such, sexual dimorphism in pituitary structure and hormonal output has been previously identified in a number of these settings (Michael et al., 1980; Lamberts and Macleod, 1990; Nishida et al., 2005). Of interest, the Drop-seq analysis of the pituitaries of 7–8 weeks old, sexually-naïve mice confirmed that even at the young adult stage there is strong evidence for sexual dimorphism. The most striking gender distinctions in this regard was the dominance of the somatotropes in the males (Fig. 5), the relative enrichment of lactotropes in in females (Fig. 5), and the distinct patterns of gonadotrope hormone expression between the two genders (Fig. 5).

In conclusion, we have combined single-cell RNA sequencing with RNA and protein single cell imaging to analyze the gene expression programs of cells within the adult mouse pituitary. The results have revealed unanticipated levels of cellular diversity and lineage plasticity in pituitary cell-type composition and hormone expression. The findings reveal a significant fraction of cells that co-express multiple hormones, sexual dimorphisms of lineage composition and cell prevalence, and the plasticity of cell functions in response to major physiologic demands. These single-cell transcriptomic data sets, along with experimental approaches to identifying the factors that underlie these complexities of lineage structure and function, can now be further extended to explore pituitary functions in settings of physiologic stress and disease.

## Methods

### Animals

Six-week old CD1 mice were purchased from Charles River (Wilmington, MA) and were housed for a minimum of 1 to 2 weeks in rooms with 12-hour light/dark cycles prior to studies. All aspects of the mouse studies were approved by the University of Pennsylvania Laboratory Animal Use and Care Committee. The *mt*/*hGRF* transgenic mice were originally obtained from Dr. Ralph Brinster at the University of Pennsylvania (Hammer et al., 1985) and have been described in a number of our prior reports (Ho et al., 2002; Ho et al., 2008).

### Single cell preparation

Single cell pituitary suspensions were prepared by non-enzymatic methods as previously descripted (Ho et al., 2011) with minor modifications to adapt to the Drop-seq protocol (Macosko et al., 2015). Briefly, the pituitaries were isolated and washed with cold PBS, incubated with 1 mL of enzyme-free cell dissociation buffer (Life technologies, Carlsbad, CA) for 1 min, passed through a 40 μm cell strainer, and then re-suspend in PBS.

### Single-cell RNA-Seq library preparation and sequencing

Drop-seq was performed as previously described with minor modifications (Macosko et al., 2015). Specifically, cells were captured on barcoded beads, reverse transcribed, and treated with exonuclease prior to amplification. The cDNA from an aliquot of 6,000 beads was amplified by PCR in a volume of 50 μL (25 μL of 2× KAPA HiFi hotstart readymix (KAPA biosystems), 0.4 μL of 100 μmol/L TSO-PCR primer (AAGCAGTGGTATCAACGCAGAGT), 24.6 μL of nuclease-free water) to determine an optimal number of PCR cycles for cDNA amplification. The thermal cycling parameter was set to: 95 °C for 3 min; 4 cycles of 98 °C for 20 s, 65 °C for 45 s, 72 °C for 3 min; 9 cycles of 98 °C for 20 s, 67 °C for 45 s, 72 °C for 3 min; 72 °C for 5 min, hold at 4 °C. The amplified cDNA was purified twice with 0.6× SPRISelect beads (Beckman Coulter) and eluted with 10 μL of water. 10% of amplified cDNA was used to perform real-time PCR analysis (1 μL of purified cDNA, 0.2 μL of 25 μmol/L TSO-PCR primer, 5 μL of 2× KAPA FAST qPCR readymix, and 3.8 μL of water) to determine the additional number of PCR cycles needed for optimal cDNA amplification (Applied Biosystems QuantStudio 7 Flex). PCR reactions were then optimized per total number of barcoded beads collected for each Drop-seq run, adding 6,000 beads per PCR tube, and run according to the aforementioned program to enrich the cDNA for 4 + 12 to 13 cycles. The amplified cDNA was ‘tagmented’ using the Nextera XT DNA sample preparation kit (Illumina, cat# FC-131-1096), starting with 550 pg of cDNA pooled in equal amounts from all PCR reactions for a given run. After quality control analysis using a Bioanalyzer (Agilent), libraries were sequenced on an Illumina NextSeq 500 instrument using the 75-cycle High Output v2 Kit (Illumina). The library was loaded at 2.0 pmol/L and the Custom Read1 Primer (GCCTGTCCGCGGAAGCAGTGGTATCAACGCAGAGTAC) was added at 0.3 μmol/L in position 7 of the reagent cartridge. The sequencing configuration was 20 bp (Read1), 8 bp (Index1), and 50 or 60 bp (Read2). Two male samples and two female samples (two mice per sample) from 8-week old CD1 mice, 1 sample from 13-week old virgin mouse, 2 samples from 13-week lactation mice and 1 sample from two *mt*/*hGRF* transgenic mice (Table S2), were analyzed with Drop-seq in five sequencing runs. For 10X Genomics platform, the single cell suspension of 8-week old mouse pituitary was loaded onto a well on a 10x Chromium Single Cell instrument. Library preparation was performed according to the manufacturer’s instructions (Chromium Single Cell 3’ Library & Gel Bead Kit v2). All sequencing data associated with this study have been deposited to Gene Expression Omnibus (GEO) under the accession code GSE146619. The analysis source code underlying the final version of the paper is available on GitHub repository (https://gibhub.com/wulabupenn/mPit).

### Read mapping

Paired-end sequencing reads of Drop-seq were processed as previous described (Hu et al., 2017). Briefly, after mapping the reads to the mouse genome (mm10, Gencode release vM13), exonic reads mapped to the predicted strands of annotated genes were retrieved for the cell type classification. Uniquely mapped reads were grouped by cell barcode. To digitally count gene transcripts, a list of UMIs in each gene, within each cell, was assembled, and UMIs within ED = 1 were merged together. The total number of unique UMI sequences was counted, and this number was reported as the number of transcripts of that gene for a given cell. Raw digital expression matrices were generated for all of the 8 samples. For 10X Genomics data sets, cellranger was used to generate digital expression matrices.

### Cell type classification

To enable directly comparative analyses among different conditions, such as male versus female, lactation versus virgin, WT versus *mt*/*hGRF* transgenic mice, we used Seurat 3 (v. 3.0.0) which has been demonstrated as an effective approach to perform joint analyses and build an integrated reference (Stuart et al., 2019) (Fig. S2A and S2B). The raw digital expression matrices of all 8 samples from Drop-seq runs were combined and loaded into the Seurat 3. For normalization, UMI counts for all cells were scaled by library size (total UMI counts), multiplied by 10,000, and transformed to log space. Only genes found to be expressing in >10 cells were retained. Cell with a high percentage of UMIs mapping to mitochondrial genes (≥0.1) were discarded. In addition, cells with fewer than 300 UMI counts, fewer than 100 detected genes, or more than 4,000 detected genes were discarded, resulting in 19,867 cells from 8 samples. The nUMIs and nGenes are shown (Fig. S2C). The top 2,000 highly variable genes (HVGs) were identified using the function *FindVariableFeatures* with “vst” method. Canonical correlation analysis (CCA) was used to identify common sources of variation among WT, *hGHRF* transgenic mice, and lactating mice. The first 30 dimensions of the CCA was chosen to integrate the 6 datasets, including 2 replicates of 8-week old WT mice, 1 replicate of 13-week old virgin female mice, 2 replicates of 13-week old lactation female mice and 1 replicate of 8-week old *mt*/*hGRF* transgenic mice. After integration, the expression levels of HVGs in the cells were scaled and centered along each gene and was subjected to PCA analysis. The top 25 PCs were selected and used for 2-dimension reduction by Uniform Manifold Approximation and Projection (UMAP). Clusters were identified using the function *FindCluster* in Seurat with the resolution parameter set to 0.4. Assessing a number of different PCs for clustering revealed that the variation of PC number selection was relatively insensitive to the clustering results (Fig. S2D). Cells were classified into 14–22 clusters with the resolution parameter from 0.4 to 1 (Fig. S2E). Clustering resolution parameters were varied quantitatively based on the number of cells being clustered. After the clustering results with different resolutions were compared and evaluated, we chose a resolution value of 0.4. Using this approach, we were able to assign 19,660 cells to 14 clusters. Two cell clusters, which was annotated as red blood cell and NK cell cluster, were excluded in the downstream analysis. We further filtered out two clusters; one cluster contained 638 cells with low quality and the other cluster, with almost double number of genes per cell as compared to other cell clusters, were considered cell doublets. In all, 1,462 cells (7.4% of input data) were removed from the downstream analysis and 18,405 cells were assigned into 10 cell clusters. Marker genes were identified using the function *FindMarkers* in Seurat. Cell type was annotated based on top ranked marker genes. To this end, the information was used to build an integrated cell type reference of mouse pituitary (Table S2).

For cell type classification of 10X Genomic data set, low quality cells were filtered out (200 < nGenes < 3000 and percent.mt < 10). Canonical correlation analysis (CCA) was used to identify common sources of variation between male and female mice and then used to integrate two data sets. After integration, the expression levels of HVGs in the cells were scaled and centered along each gene and was subjected to PCA analysis. The top 15 PCs were selected and used for 2-dimension reduction by Uniform Manifold Approximation and Projection (UMAP). Clusters were identified using the function *FindCluster* in Seurat with the resolution parameter set to 0.4. Clustering resolution parameters were varied quantitatively based on the number of cells being clustered. After the clustering results with different resolutions were compared and evaluated, we chose a relative high-resolution value of 1.6. After checking the top marker expression in each cluster, we empirically merged clusters showing largely overlap of top marker genes and finally were able to assign 2,780 cells to 10 clusters.

### Background correction of the cell transcriptomes

After clustering, we found that highly transcribed hormone genes (*Gh*, *Prl* and *Pomc*) could be detected in blood cells. This observation most likely represented cross-contamination of free RNAs, which is a common issue for all the droplet based single cell analysis (Cheung et al., 2018; Fletcher et al., 2019). Since these mRNAs are highly expressed in the pituitary but should be absent in the blood cells, their levels in blood cells represent a background noise signals. We adapted a previous background correction approach (Han et al., 2018; Fletcher et al., 2019) to correct the level of contaminated mRNA. We defined expression threshold of contaminated genes as cutoff of background noise. The relative expression level of highly expressed genes (Gh, Prl and Pomc) were then shown in the dot plot (Figs. 2C and S2A). To evaluate different method of ambient RNA correction, we adopted SoupX (Young and Behjati, 2018) to correct the ambient RNA of each library and the results showed the consistence between two methods.

### Comparison of single cell RNA-seq data sets

To assess the validity of single cell RNA-seq results between 2 platforms or this study with previous study (Cheung et al., 2018). To compare cell-type-specific expression signatures, we computed the pairwise Pearson correlation coefficients between each pair of cell types in the data set for a common set of genes. To generate a common marker gene list for the pairwise comparison, we identified marker genes for different single cell data set by function *FindAllMarkers* in R package Seurat, respectively. Average natural log scaled UMI counts were used to generate the gene expression matrix, respectively. R function cor.test was used to calculate the pairwise Pearson correlation coefficients.

### Identification of differentially expressed genes

Differential gene expression analysis between control mice and *Ghrh* transgenic mice or between control mice and lactating mice was performed using the function *FindMarkers* in Seurat, using a Wilcoxon rank sum test. Genes with an adjust *P*-value less than 0.05 were considered to be differentially expressed. For Table S3, we ranked the genes by adjust *P*-value. Top 100 genes of each cell cluster were subjected to gene ontology (GO) enrichment analysis. GO enrichment analysis were performed as previous (Hu et al., 2018). The significance of enriched GO term was assessed by hypergeometric distribution.

### Probe design for single-cell RNA fluorescent *in situ* hybridization (scRNA FISH)

The strategy for the scRNA FISH probe design followed the strategy generally used for Single-molecule Oligopaint FISH studies (Beliveau et al., 2015). Each 40 nt primary probe contained 20 nt complementary to the exons of the mRNAs (*Gh*, *Prl*, and *POMC)* with a 20 nt non-genomic tag located at the 5′ end. 20 distinct primary probes scanning each targeted mRNA were used in each probe library. The tag sequence was unique for each mRNA library (Beliveau et al., 2015). A fluorophore-labeled secondary oligo with base complementarity to the tag sequence was used to detect the hybridized primary probes. All oligos were synthesized by Integrated DNA Technologies (IDT; Coralville, IA). The sequences of the primary and secondary oligos are available upon request.

### Preparation of pituitary tissue sections

The pituitaries of 8-week old mice were excised and washed in ice-cold PBS. The tissue was fixed in 4% paraformaldehyde for 2 h at 4 °C. The tissue was cryo-protected in 30% sucrose and embedded in Tissue-Tek OCT compound (Sakyra Finetek USA Inc, REF:4583) and frozen at −80 °C. Coronal or sagittal sections (6 μm thickness) were generated and mounted on slides. The slides were dried at room temperature for 2 h and stored at −20 °C prior to analysis.

### scRNA FISH and immuno-fluorescent-scRNA (IF-scRNA) FISH

The procedures for RNA FISH were as described (Ragoczy et al., 2006; Ho et al., 2013; Beliveau et al., 2015) with modifications. Briefly, slides were removed from −20 °C storage and dried at room temperature for 1 h. The slides were washed with PBS, fixed with 3.7% formaldehyde in PBS for 20 min, washed with PBS and treated with 70% ethanol at 4 °C for overnight. 500 pmol of primary oligo library (19 to 22 oligos) and equal amount of fluorophore-labeled secondary were used for each slide (6–8 tissue sections). Prior to hybridization, the slides were washed with PBS, sequentially dehydrated in 70%, 90%, and 100% ETOH, and then equilibrated in 10% formamide/2× SSC, pH 7.0. The mixture of the primary oligo probes and the secondary probes were hybridized to the cells in 10% formamide/10% dextran sulfate/2× SSC/5 mmol/L ribonucleotide vanadate complex/0.05% BSA/1 μg/μL *E*. *coli* tRNA. The probes were heat denatured at 85 °C for 5 min, pre-annealed at 37 °C, and then hybridized overnight at 37 °C in a humidified chamber. Slides were sequentially washed in 10% formamide/2× SSC, pH 7 followed by 2× SSC at 37 °C and then mounted with Fluoroshield with DAPI (Sigma, St. Louis, MO).

For IF analysis, the slides were removed from −20°C, dried at room temperature for 1 h, washed with PBS containing 0.1% Triton X-100 (PBST) (3 × 15 min), incubated with blocking buffer (2% BSA, 0.1% Triton X-100, and 5% normal donkey serum in PBS) for 1 h and then incubated with primary antibodies for overnight at 4 °C. GH was detected using monkey anti-rat GH that cross-reacts with mGh. PRL was detected using rabbit anti-mouse PRL (National Hormone and Peptide Program, NIH) or goat anti-PRL antibody (ThermoFisher Scientific, PA5-47140). ACTH and TSHβ were detected using rabbit-anti-ACTH antibody or TSHβ antibody, respectively (National Hormone and Peptide Program, NIH). The secondary antibodies used were donkey anti-human, anti-rabbit, anti-goat antibodies (Jackson immnoResearch Inc). The slides were mounted as for the scRNA FISH.

For the combined IF/scRNA FISH studies, the RNA FISH was performed as described above and the slides were then equilibrated in PBST for 10 min at room temperature, blocked with blocking buffer containing 2 mmol/L ribonucleotide vanadate complex, and IF was performed as described above.

For the scRNA FISH and IF studies of disassociated single cells, the pituitaries were disassociated as described in the Drop-seq procedure followed by fixation in poly-lysine coated slides and the procedures for RNA and protein analyses were then performed as described above.

### Image analysis

Image capture were collected on a Leica TCS SP8 confocal microscope platform. The images were analyzed using ImageJ software (NIH).

## Electronic supplementary material

Below is the link to the electronic supplementary material.

Supplementary material 1 (PDF 45585 kb)

Supplementary material 2 (XLSX 500 kb)

## References

[CR1] Andersen B, Pearse RV, Jenne K, Sornson M, Lin SC, Bartke A, Rosenfeld MG (1995). The Ames dwarf gene is required for Pit-1 gene activation. Dev Biol.

[CR2] Andoniadou CL, Matsushima D, Mousavy Gharavy SN, Signore M, Mackintosh AI, Schaeffer M, Gaston-Massuet C, Mollard P, Jacques TS, Le Tissier P (2013). Sox2(+) stem/progenitor cells in the adult mouse pituitary support organ homeostasis and have tumor-inducing potential. Cell Stem Cell.

[CR3] Barbosa JA, Gill BM, Takiyyuddin MA, O’Connor DT (1991). Chromogranin A: posttranslational modifications in secretory granules. Endocrinology.

[CR4] Beliveau BJ, Boettiger AN, Avendano MS, Jungmann R, McCole RB, Joyce EF, Kim-Kiselak C, Bantignies F, Fonseka CY, Erceg J (2015). Single-molecule super-resolution imaging of chromosomes and in situ haplotype visualization using Oligopaint FISH probes. Nat Commun.

[CR5] Budry L, Balsalobre A, Gauthier Y, Khetchoumian K, L’Honore A, Vallette S, Brue T, Figarella-Branger D, Meij B, Drouin J (2012). The selector gene Pax7 dictates alternate pituitary cell fates through its pioneer action on chromatin remodeling. Genes Dev.

[CR6] Campbell JN, Macosko EZ, Fenselau H, Pers TH, Lyubetskaya A, Tenen D, Goldman M, Verstegen AM, Resch JM, McCarroll SA (2017). A molecular census of arcuate hypothalamus and median eminence cell types. Nat Neurosci.

[CR7] Camper SA, Saunders TL, Katz RW, Reeves RH (1990). The Pit-1 transcription factor gene is a candidate for the murine Snell dwarf mutation. Genomics.

[CR8] Cao D, Ma X, Cai J, Luan J, Liu AJ, Yang R, Cao Y, Zhu X, Zhang H, Chen YX (2016). ZBTB20 is required for anterior pituitary development and lactotrope specification. Nat Commun.

[CR9] Castrique E, Fernandez-Fuente M, Le Tissier P, Herman A, Levy A (2010). Use of a prolactin-Cre/ROSA-YFP transgenic mouse provides no evidence for lactotroph transdifferentiation after weaning, or increase in lactotroph/somatotroph proportion in lactation. J Endocrinol.

[CR10] Cawley NX, Li Z, Loh YP (2016). 60 YEARS OF POMC: biosynthesis, trafficking, and secretion of pro-opiomelanocortin-derived peptides. J Mol Endocrinol.

[CR11] Chen R, Wu X, Jiang L, Zhang Y (2017). Single-cell RNA-seq reveals hypothalamic cell diversity. Cell Rep.

[CR12] Cheung LYM, George AS, McGee SR, Daly AZ, Brinkmeier ML, Ellsworth BS, Camper SA (2018). Single-cell RNA sequencing reveals novel markers of male pituitary stem cells and hormone-producing cell types. Endocrinology.

[CR13] Childs GV (2000). Green fluorescent proteins light the way to a better understanding of the function and regulation of specific anterior pituitary cells. Endocrinology.

[CR14] Davis SW, Ellsworth BS, Perez Millan MI, Gergics P, Schade V, Foyouzi N, Brinkmeier ML, Mortensen AH, Camper SA (2013). Pituitary gland development and disease: from stem cell to hormone production. Curr Top Dev Biol.

[CR15] Davis SW, Keisler JL, Perez-Millan MI, Schade V, Camper SA (2016). All hormone-producing cell types of the pituitary intermediate and anterior lobes derive from Prop1-expressing progenitors. Endocrinology.

[CR16] Devnath S, Inoue K (2008). An insight to pituitary folliculo-stellate cells. J Neuroendocrinol.

[CR17] Durand D, Pampillo M, Caruso C, Lasaga M (2008). Role of metabotropic glutamate receptors in the control of neuroendocrine function. Neuropharmacology.

[CR18] Fletcher PA, Smiljanic K, Maso Prévide R, Iben J, Li T, Rokic MB, Sherman A, Coon SL, Stojilkovic SS (2019). Cell type-and sex-dependent transcriptome profiles of rat anterior pituitary cells. Front Endocrinol.

[CR19] Frawley LS, Boockfor FR (1991). Mammosomatotropes: presence and functions in normal and neoplastic pituitary tissue. Endocr Rev.

[CR20] Gage PJ, Brinkmeier ML, Scarlett LM, Knapp LT, Camper SA, Mahon KA (1996). The Ames dwarf gene, df, is required early in pituitary ontogeny for the extinction of Rpx transcription and initiation of lineage-specific cell proliferation. Mol Endocrinol.

[CR21] Gaylinn BD (2002). Growth hormone releasing hormone receptor. Recept Channels.

[CR22] Gill BM, Barbosa JA, Dinh TQ, Garrod S, O’Connor DT (1991). Chromogranin B: isolation from pheochromocytoma, N-terminal sequence, tissue distribution and secretory vesicle processing. Regul Pept.

[CR23] Hammer RE, Brinster RL, Rosenfeld MG, Evans RM, Mayo KE (1985). Expression of human growth hormone-releasing factor in transgenic mice results in increased somatic growth. Nature.

[CR24] Han X, Wang R, Zhou Y, Fei L, Sun H, Lai S, Saadatpour A, Zhou Z, Chen H, Ye F (2018). Mapping the mouse cell atlas by microwell-seq. Cell.

[CR25] Ho Y, Elefant F, Cooke N, Liebhaber S (2002). A defined locus control region determinant links chromatin domain acetylation with long-range gene activation. Mol Cell.

[CR26] Ho Y, Tadevosyan A, Liebhaber SA, Cooke NE (2008). The juxtaposition of a promoter with a locus control region transcriptional domain activates gene expression. EMBO Rep.

[CR71] Ho Y, Liebhaber SA, Cooke NE (2011). The role of the hGH locus control region in somatotrope restriction of hGH-N gene expression. Mol Endocrinol.

[CR27] Ho Y, Shewchuk BM, Liebhaber SA, Cooke NE (2013). Distinct chromatin configurations regulate the initiation and the maintenance of hGH gene expression. Mol Cell Biol.

[CR28] Hu P, Fabyanic E, Kwon DY, Tang S, Zhou Z, Wu H (2017). Dissecting cell-type composition and activity-dependent transcriptional state in mammalian brains by massively parallel single-nucleus RNA-seq. Mol Cell.

[CR30] Hu P, Liu J, Zhao J, Wilkins BJ, Lupino K, Wu H, Pei L (2018). Single-nucleus transcriptomic survey of cell diversity and functional maturation in postnatal mammalian hearts. Genes Dev.

[CR31] Ingraham HA, Lala DS, Ikeda Y, Luo X, Shen WH, Nachtigal MW, Abbud R, Nilson JH, Parker KL (1994). The nuclear receptor steroidogenic factor 1 acts at multiple levels of the reproductive axis. Genes Dev.

[CR32] Janknecht R (2010). Multi-talented DEAD-box proteins and potential tumor promoters: p68 RNA helicase (DDX5) and its paralog, p72 RNA helicase (DDX17). Am J Transl Res.

[CR33] Kelberman D, Rizzoti K, Lovell-Badge R, Robinson IC, Dattani MT (2009). Genetic regulation of pituitary gland development in human and mouse. Endocr Rev.

[CR34] Lamberts SW, Macleod RM (1990). Regulation of prolactin secretion at the level of the lactotroph. Physiol Rev.

[CR35] Lamolet B, Pulichino AM, Lamonerie T, Gauthier Y, Brue T, Enjalbert A, Drouin J (2001). A pituitary cell-restricted T box factor, Tpit, activates POMC transcription in cooperation with Pitx homeoproteins. Cell.

[CR36] Le Tissier PR, Hodson DJ, Martin AO, Romano N, Mollard P (2015). Plasticity of the prolactin (PRL) axis: mechanisms underlying regulation of output in female mice. Adv Exp Med Biol.

[CR37] Li S, Crenshaw EB, Rawson EJ, Simmons DM, Swanson LW, Rosenfeld MG (1990). Dwarf locus mutants lacking three pituitary cell types result from mutations in the POU-domain gene pit-1. Nature.

[CR38] Lin SC, Lin CR, Gukovsky I, Lusis AJ, Sawchenko PE, Rosenfeld MG (1993). Molecular basis of the little mouse phenotype and implications for cell type-specific growth. Nature.

[CR39] Liu NA, Liu Q, Wawrowsky K, Yang Z, Lin S, Melmed S (2006). Prolactin receptor signaling mediates the osmotic response of embryonic zebrafish lactotrophs. Mol Endocrinol.

[CR40] Macosko EZ, Basu A, Satija R, Nemesh J, Shekhar K, Goldman M, Tirosh I, Bialas AR, Kamitaki N, Martersteck EM (2015). Highly parallel genome-wide expression profiling of individual cells using nanoliter droplets. Cell.

[CR41] Mayo KE, Hammer RE, Swanson LW, Brinster RL, Rosenfeld MG, Evans RM (1988). Dramatic pituitary hyperplasia in transgenic mice expressing a human growth hormone-releasing factor gene. Mol Endocrinol.

[CR42] Mayo KE, Miller T, DeAlmeida V, Godfrey P, Zheng J, Cunha SR (2000). Regulation of the pituitary somatotroph cell by GHRH and its receptor. Recent Prog Horm Res.

[CR43] Mayran A, Khetchoumian K, Hariri F, Pastinen T, Gauthier Y, Balsalobre A, Drouin J (2018). Pioneer factor Pax7 deploys a stable enhancer repertoire for specification of cell fate. Nat Genet.

[CR44] Michael SD, Kaplan SB, Macmillan BT (1980). Peripheral plasma concentrations of LH, FSH, prolactin and GH from birth to puberty in male and female mice. J Reprod Fertil.

[CR45] Nakajima T, Yamaguchi H, Takahashi K (1980). S100 protein in folliculostellate cells of the rat pituitary anterior lobe. Brain Res.

[CR46] Nakane PK (1970). Classifications of anterior pituitary cell types with immunoenzyme histochemistry. J Histochem Cytochem.

[CR47] Nishida Y, Yoshioka M, St-Amand J (2005). Sexually dimorphic gene expression in the hypothalamus, pituitary gland, and cortex. Genomics.

[CR48] Ohta K, Nobukuni Y, Mitsubuchi H, Fujimoto S, Matsuo N, Inagaki H, Endo F, Matsuda I (1992). Mutations in the Pit-1 gene in children with combined pituitary hormone deficiency. Biochem Biophys Res Commun.

[CR49] Peel MT, Ho Y, Liebhaber SA (2018). Transcriptome analyses of female somatotropes and lactotropes reveal novel regulators of cell identity in the pituitary. Endocrinology.

[CR50] Perez Millan MI, Brinkmeier ML, Mortensen AH, Camper SA (2016). PROP1 triggers epithelial-mesenchymal transition-like process in pituitary stem cells. Elife.

[CR51] Philips A, Maira M, Mullick A, Chamberland M, Lesage S, Hugo P, Drouin J (1997). Antagonism between Nur77 and glucocorticoid receptor for control of transcription. Mol Cell Biol.

[CR52] Radovick S, Nations M, Du Y, Berg LA, Weintraub BD, Wondisford FE (1992). A mutation in the POU-homeodomain of Pit-1 responsible for combined pituitary hormone deficiency. Science.

[CR53] Ragoczy T, Bender MA, Telling A, Byron R, Groudine M (2006). The locus control region is required for association of the murine beta-globin locus with engaged transcription factories during erythroid maturation. Genes Dev.

[CR54] Rizzoti K, Akiyama H, Lovell-Badge R (2013). Mobilized adult pituitary stem cells contribute to endocrine regeneration in response to physiological demand. Cell Stem Cell.

[CR55] Seuntjens E, Hauspie A, Roudbaraki M, Vankelecom H, Denef C (2002). Combined expression of different hormone genes in single cells of normal rat and mouse pituitary. Arch Physiol Biochem.

[CR56] Seuntjens E, Hauspie A, Vankelecom H, Denef C (2002). Ontogeny of plurihormonal cells in the anterior pituitary of the mouse, as studied by means of hormone mRNA detection in single cells. J Neuroendocrinol.

[CR57] Shekhar K, Lapan SW, Whitney IE, Tran NM, Macosko EZ, Kowalczyk M, Adiconis X, Levin JZ, Nemesh J, Goldman M (2016). Comprehensive classification of retinal bipolar neurons by single-cell transcriptomics. Cell.

[CR58] Smith MS, Fox SR (1984). Regulation of gonadotropin secretion during lactation. Arch Biol Med Exp.

[CR59] Stuart T, Satija R (2019). Integrative single-cell analysis. Nat Rev Genet.

[CR60] Stuart T, Butler A, Hoffman P, Hafemeister C, Papalexi E, Mauck WM, Hao Y, Stoeckius M, Smibert P, Satija R (2019). Comprehensive integration of single-cell data. Cell.

[CR61] Tanay A, Regev A (2017). Scaling single-cell genomics from phenomenology to mechanism. Nature.

[CR62] Theogaraj E, John CD, Christian HC, Morris JF, Smith SF, Buckingham JC (2005). Perinatal glucocorticoid treatment produces molecular, functional, and morphological changes in the anterior pituitary gland of the adult male rat. Endocrinology.

[CR63] Vazquez-Borrego MC, Gahete MD, Martinez-Fuentes AJ, Fuentes-Fayos AC, Castano JP, Kineman RD, Luque RM (2018). Multiple signaling pathways convey central and peripheral signals to regulate pituitary function: lessons from human and non-human primate models. Mol Cell Endocrinol.

[CR64] Villalobos C, Nunez L, Garcia-Sancho J (2004). Anterior pituitary thyrotropes are multifunctional cells. Am J Physiol Endocrinol Metab.

[CR65] Villalobos C, Nunez L, Garcia-Sancho J (2004). Phenotypic characterization of multi-functional somatotropes, mammotropes and gonadotropes of the mouse anterior pituitary. Pflugers Arch.

[CR66] Young MD, Behjati S (2018). SoupX removes ambient RNA contamination from droplet based single cell RNA sequencing data. BioRxiv.

[CR67] Zahuczky G, Kristof E, Majai G, Fesus L (2011). Differentiation and glucocorticoid regulated apopto-phagocytic gene expression patterns in human macrophages. Role of Mertk in enhanced phagocytosis. PLoS ONE.

[CR68] Zheng GX, Terry JM, Belgrader P, Ryvkin P, Bent ZW, Wilson R, Ziraldo SB, Wheeler TD, McDermott GP, Zhu J (2017). Massively parallel digital transcriptional profiling of single cells. Nat Commun.

[CR69] Zhu X, Gleiberman AS, Rosenfeld MG (2007). Molecular physiology of pituitary development: signaling and transcriptional networks. Physiol Rev.

[CR70] Zhu X, Tollkuhn J, Taylor H, Rosenfeld MG (2015). Notch-dependent pituitary SOX2(+) stem cells exhibit a timed functional extinction in regulation of the postnatal gland. Stem Cell Rep.

