# Supplementary material for: Single-cell transcriptomic analysis of adult mouse pituitary reveals sexual dimorphism and physiologic demand-induced cellular plasticity
Source: Protein Cell. 2020 Mar 19;11(8):565–83. doi: 10.1007/s13238-020-00705-x (PMC7381518; doi:10.1007/s13238-020-00705-x)
Supplement: Supplementary file 1 — Supplementary material 1 (PDF 45585 kb) [file 13238_2020_705_MOESM1_ESM.pdf]

Supplemental Figure Legends

Figure S1. Standard model of anterior pituitary development and lineage specification
This diagram summarizes the standard model of pituitary lineage differentiation. The development of the mouse anterior pituitary from the oral ectoderm is driven by a complex array of signaling pathways and transcription factors, a subset of which are indicated. The adult pituitary is characterized by the presence of six terminally differentiated cell types, each defined by the expression of its corresponding hormone (Prl, prolactin; Gh, growth hormone; Tshb; β subunit of thyroid stimulating hormone; Lhβ and Fshβ, the β subunits of the luteinizing and follicle stimulating hormones; POMC, proopiomelanocortin prohormone; ACTH, adrenocorticotropic hormone; α-MSH, melanocyte stimulating hormone). Differentiation of three lineages (lactotropes, somatotropes and thyrotropes) is specifically driven by, and dependent on, the POU-homeo domain transcription factor, Pou1f1. The remaining three lineages (corticotropes, melanotropes, and gonadotropes) are considered to be Pou1f1-independent.

Figure S2. Cell type classification and comparison with data from the present and prior studies

- **A.** Dot plot of normalized expression level of top markers of each cluster.
- **B.** Heatmap of scaled one-to-one correlation efficient of cell type clusters of 8-wk WT mice between 2 single cell platforms. The asterisks indicate the best matched cell type between the two data sets.
- **C.** Our UMAP visualization of published data (Cheung, 2018) of 10,248 pituitary cells from seven-week-old C57BL/6 male mice analyzed by 10 x Genomics platform.
- **D.** Heatmap of scaled one-to-one correlation efficient of cell type clusters between the two 10 x Genomics studies. The asterisks indicate the best matched cell type between the two studies.

Figure S3. Seurat integration of 18,405 pituitary cells from Dop-seq experiments.

Figure S4. RNA FISH and IF analyses in adult pituitary tissue sections detect the presence of the multi-hormone cells at both mRNA and protein levels.
A. RNA FISH demonstrates that the morphology of the adult pituitary is conserved in pituitary tissue sections. This analysis was performed on pituitary tissue sections of 8-week old male and female mice.

Left panel: RNA FISH shows that the robust expression of the *Prl* mRNA (green) is restricted to the anterior lobe (AL).

Middle panel: RNA FISH shows the robust expression of the *Gh* mRNA (red) is restricted to the anterior lobe (AL).

Right panel: RNA FISH shows the broad expression of *Pomc* mRNA (grey) in the intermediate lobe (IL) and anterior lobe (AL).

B. IF analysis of male and female adult mouse pituitary tissue sections reveals sexual dimorphism for FSHβ expression *in vivo.*

Left panel: FSH**β** protein (red) as detected by anti-FSHβ antibody in the pituitary tissue sections of male mice. Scale bar = 5 μm.

Right panel: FSH**β** protein (red) as detected by anti-FSHβ antibody in the pituitary tissue sections of female mice. Scale bar = 5 μm.

Histogram: analysis of male and female pituitary cells. Pituitary tissue sections from 8-week old, sexually naïve male mice (n=2) were analyzed by IF. The histogram represents the percent of the total cell population positive for FSHβ in male pituitary (18.6%) and female pituitary (8.8%).

C. **IF analysis identifies cells co-expressing of TSHβ and GH proteins *in vivo*.**

IF was performed in the pituitary tissue sections of an 8-week old male mouse using antibodies specifically for GH (red in the left panel) and TSH β (green in the middle panel). The right panel is the merged IF images. Two TSHβ positive cells are indicated with arrows. One of these cells is also expressing high level GH (white arrow) while the other cell is expressing relative lower level of GH (yellow arrow).

Histogram: Quantification of cells co-expressing TSHβ and GH proteins. Of 64 TSH(+) cells detected in this study, 39 were also positive for GH (61%).

Table S1. Summary of single cell RNA-seq data sets.

Table S2. List of marker genes in each cluster.

Table S3. Enriched GO terms of top 100 differentially expressed genes in lactation or transgenic mouse pitutiary cell clusters

Figure S1

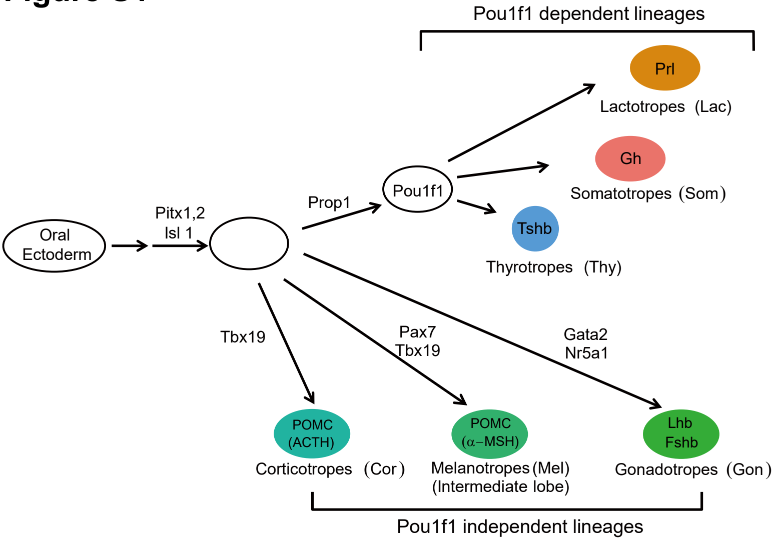

Pou1f1 dependent lineages
Prl
Lactotropes (Lac)
Gh
Somatotropes (Som)
Tshb
Thyrotropes (Thy)
Pou1f1
Prop1
Oral Ectoderm
Pitx1,2
Isl 1
Tbx19
Pax7
Tbx19
Gata2
Nr5a1
POMC
(ACTH)
Corticotropes (Cor)
POMC
(α−MSH)
Melanotropes (Mel)
(Intermediate lobe)
Lhb
Fshb
Gonadotropes (Gon)
Pou1f1 independent lineages

Figure S2

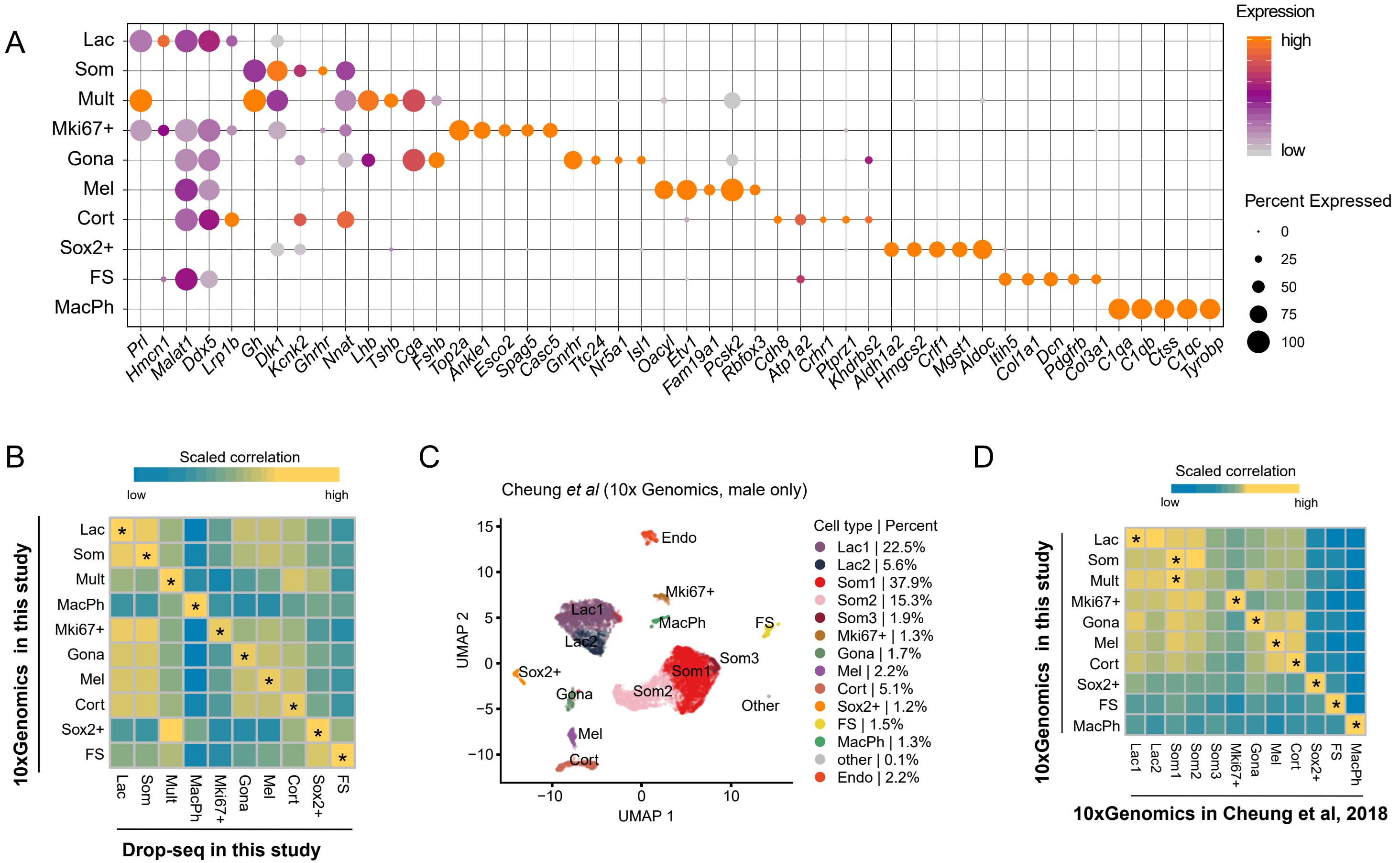
A
Expression
high
low
Percent Expressed
0
25
50
75
100
Lac
Som
Mult
Mki67+
Gona
Mel
Cort
Sox2+
FS
MacPh
Prl Hmcn1 Malat1 Ddx5 Lrp1b Gh Dlk1 Kcnk2 Ghrhr Nnat Lhb Tshb Cga Fshb Top2a Ankle1 Esco2 Spag5 Casc5 Gnrhr Ttc24 Nr5a1 Isl1 Oacyl Etv1 Fam19a1 Pcsk2 Rbfox3 Cdh8 Atp1a2 Crhr1 Ptprz1 Khdrbs2 Aldh1a2 Hmgcs2 Crlf1 Mgst1 Aldoc Itih5 Col1a1 Dcn Pdgfrb Col3a1 C1qa C1qb Ctss C1qc Tyrobp
B
Scaled correlation
low
high
10xGenomics in this study
Drop-seq in this study
C
Cheung et al (10x Genomics, male only)
UMAP 2
UMAP 1
Cell type | Percent
Lac1 | 22.5%
Lac2 | 5.6%
Som1 | 37.9%
Som2 | 15.3%
Som3 | 1.9%
Mki67+ | 1.3%
Gona | 1.7%
Mel | 2.2%
Cort | 5.1%
Sox2+ | 1.2%
FS | 1.5%
MacPh | 1.3%
other | 0.1%
Endo | 2.2%
Endo
Other
D
Scaled correlation
low
high
10xGenomics in this study
10xGenomics in Cheung et al, 2018
Lac1 Lac2 Som1 Som2 Som3 Mki67+ Gona Mel Cort Sox2+ FS MacPh

Figure S3

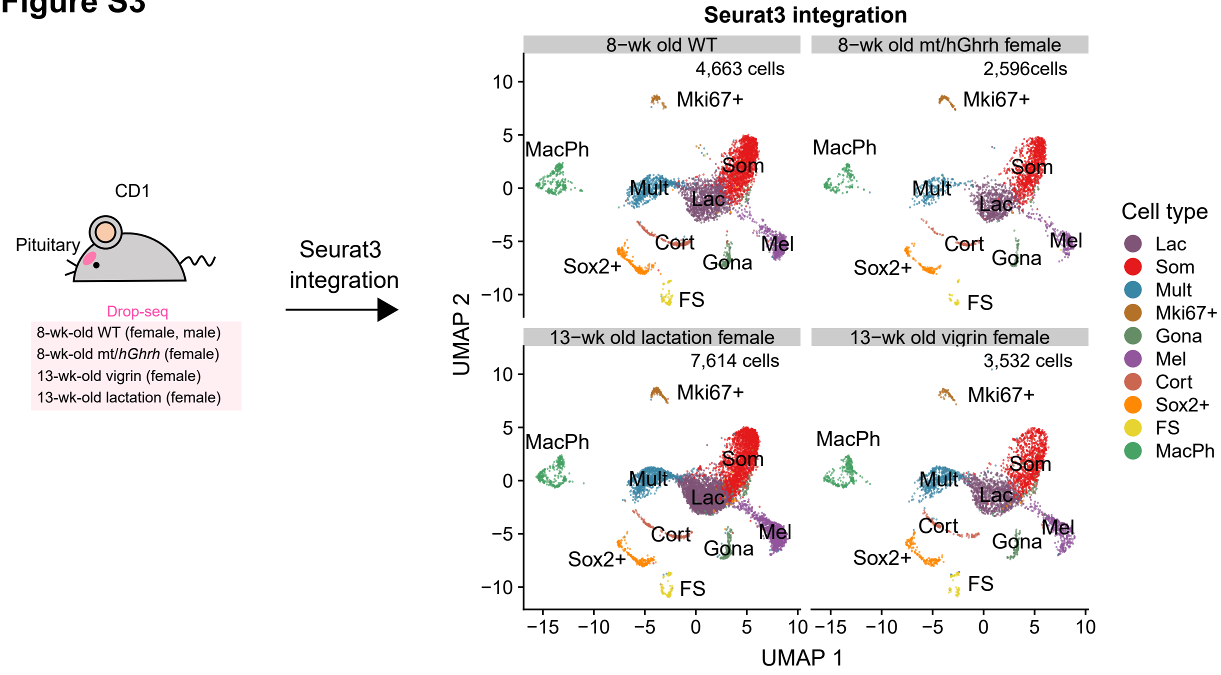

CD1
Pituitary
Drop-seq
8-wk-old WT (female, male)
8-wk-old mt/*hGhrh* (female)
13-wk-old vigrin (female)
13-wk-old lactation (female)
Seurat3 integration
Seurat3 integration
8−wk old WT
4,663 cells
8−wk old mt/hGhrh female
2,596cells
13−wk old lactation female
7,614 cells
13−wk old vigrin female
3,532 cells
Mki67+
MacPh
Som
Mult
Lac
Cort
Mel
Sox2+
Gona
FS
UMAP 2
UMAP 1
Cell type
Lac
Som
Mult
Mki67+
Gona
Mel
Cort
Sox2+
FS
MacPh

Figure S4

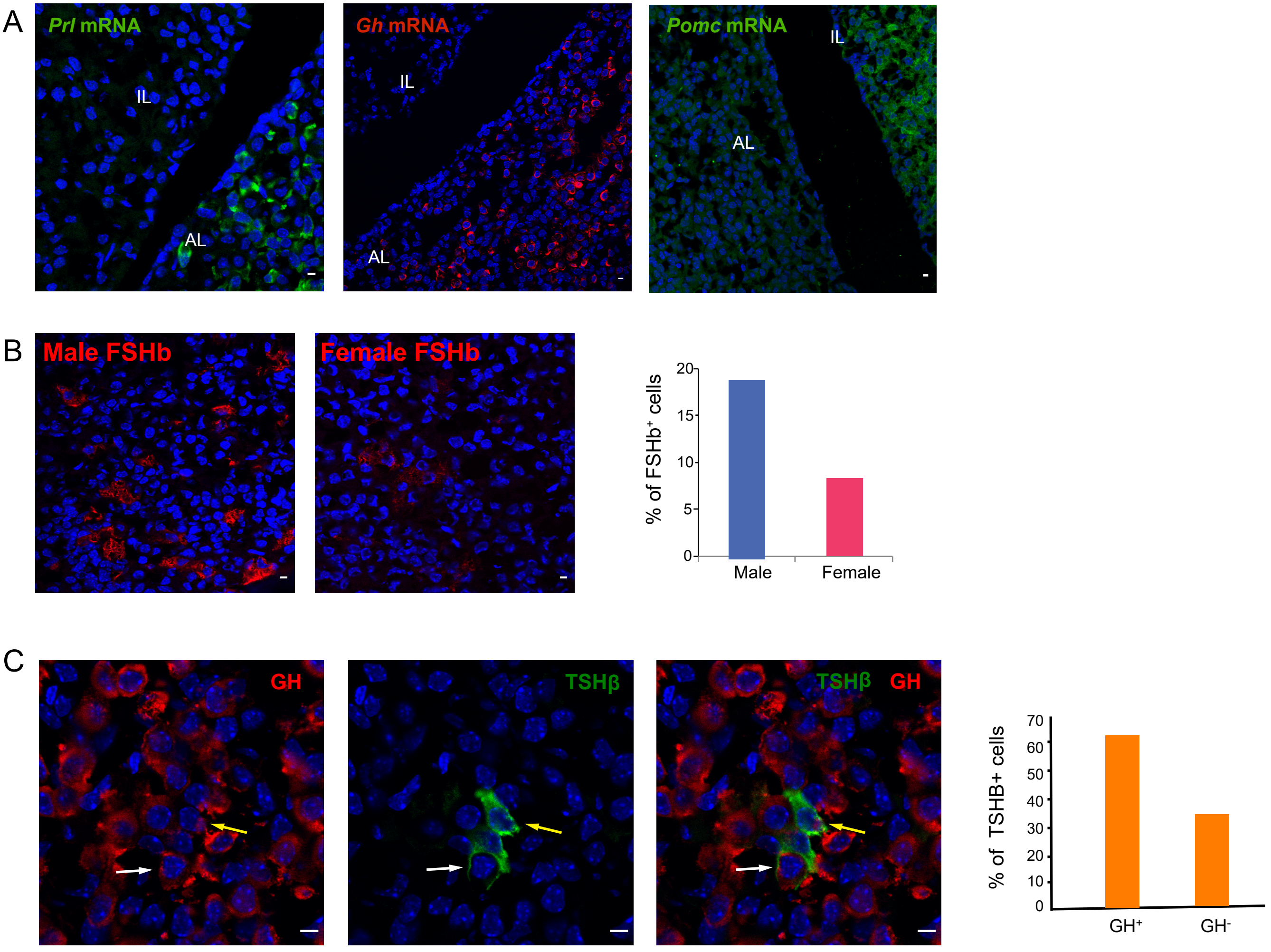
A
Prl mRNA
IL
AL
Gh mRNA
IL
AL
Pomc mRNA
IL
AL
B
Male FSHb
Female FSHb
% of FSHb+ cells
20
15
10
5
0
Male
Female
C
GH
TSHβ
TSHβ GH
% of TSHB+ cells
70
60
50
40
30
20
10
0
GH+
GH-
